# Activity-Guided Characterization of COX-2 Inhibitory Compounds in *Waltheria indica* L. Extracts

**DOI:** 10.3390/molecules26237240

**Published:** 2021-11-29

**Authors:** Michael Termer, Christophe Carola, Andrew Salazar, Cornelia M. Keck, Juergen Hemberger, Joerg von Hagen

**Affiliations:** 1 Department of Pharmaceutics and Biopharmaceutics, Philipps-University of Marburg, Robert-Koch-Str. 4, 35032 Marburg, Germany; cornelia.keck@pharmazie.uni-marburg.de; 2 Merck KGaA, Frankfurterstr. 250, 64293 Darmstadt, Germany; christophe.carola@merckgroup.com (C.C.); andrew.salazar@merckgroup.com (A.S.); joerg.von.hagen@merckgroup.com (J.v.H.); 3 Department of Life Science Engineering, Institute for Biochemical Engineering & Analytics, University of Applied Sciences, Wiesenstr. 14, 35390 Giessen, Germany; juergen.hemberger@lse.thm.de

**Keywords:** *Waltheria indica* L., tiliroside, anti-inflammation, cyclooxygenase (COX), secondary metabolites, alpha-linolenic acid, linoleic acid, oleic acid, centrifugal partition chromatography (CPC)

## Abstract

Inflammation is the body’s response to infection or tissue injury in order to restore and maintain homeostasis. Prostaglandin E2 (PGE-2) derived from arachidonic acid (AA), via up-regulation of cyclooxygenase-2 (COX-2), is a key mediator of inflammation and can also be induced by several other factors including stress, chromosomal aberration, or environmental factors. Targeting prostaglandin production by inhibiting COX-2 is hence relevant for the successful resolution of inflammation. *Waltheria indica* L. is a traditional medicinal plant whose extracts have demonstrated COX-2 inhibitory properties. However, the compounds responsible for the activity remained unknown. For the preparation of extracts with effective anti-inflammatory properties, characterization of these substances is vital. In this work, we aimed to address this issue by characterizing the substances responsible for the COX-2 inhibitory activity in the extracts and generating prediction models to quantify the COX-2 inhibitory activity without biological testing. For this purpose, an extract was separated into fractions by means of centrifugal partition chromatography (CPC). The inhibitory potential of the fractions and extracts against the COX-2 enzyme was determined using a fluorometric COX-2 inhibition assay. The characterizations of compounds in the fractions with the highest COX-2 inhibitory activity were conducted by high resolution mass spectrometry (HPLC-MS/MS). It was found that these fractions contain alpha-linolenic acid, linoleic acid and oleic acid, identified and reported for the first time in *Waltheria indica* leaf extracts. After analyzing their contents in different *Waltheria indica* extracts, it could be demonstrated that these fatty acids are responsible for up to 41% of the COX-2 inhibition observed with *Waltheria indica* extract. Additional quantification of secondary metabolites in the extract fractions revealed that substances from the group of steroidal saponins and triterpenoid saponins also contribute to the COX-2 inhibitory activity. Based on the content of compounds contributing to COX-2 inhibition, two mathematical models were successfully developed, both of which had a root mean square error (RMSE) = 1.6% COX-2 inhibitory activity, demonstrating a high correspondence between predicted versus observed values. The results of the predictive models further suggested that the compounds contribute to COX-2 inhibition in the order linoleic acid > alpha linolenic acid > steroidal saponins > triterpenoid saponins. The characterization of substances contributing to COX-2 inhibition in this study enables a more targeted development of extraction processes to obtain *Waltheria indica* extracts with superior anti-inflammatory properties.

## 1. Introduction

Inflammation is a defensive response of the body to various stimuli and is a cyclic, self-stimulating process designed to combat infection or tissue injury [1,2]. The inflammatory process is accompanied by the release of pro-inflammatory cytokines and prostaglandins, and the formation of reactive oxygen species (ROS) [3]. Important modulators of inflammation are nuclear factor kappa B (NF-kB), lipoxygenase (LOX) and cyclooxygenase (COX), with NF-kB activating the expression of LOX or COX [4,5]. Both enzymes are part of the arachidonic acid (AA) metabolism, one of the main cellular processes for mediating inflammation, where they exhibit a catalytic effect. The COX-1 and COX-2 enzymes are isozymes, and in normal human skin, COX-1 is present through the epidermis, whereas COX-2 localizes mainly in suprabasal keratinocytes [6,7,8]. While COX-1 is involved in homoeostatic processes and expressed constitutively in most tissues, the pro-inflammatory COX-2 is an inducible isoform and is mainly produced in inflamed tissues. To maintain and restore homeostasis of the skin, different cellular components regulate local immune responses thorough crosstalk [2,9]. The initiation and the maintenance of the inflammation is carried out by pro-inflammatory mediators. Once the instigating factor is removed, activity is balanced out by the anti-inflammatory mediators responsible for limiting the inflammation [10,11]. Several factors including stress, chromosomal aberration or environmental factors disturb this balance and lead to excessive production of prostaglandin E2 (PGE2) derived from arachidonic acid, via the up-regulation of COX-2, consequently leading to inflammatory-mediated diseases [12,13,14,15,16].

Increased PGE2 levels lead to increased production of proinflammatory cytokines such as interleukin-6 (IL-6), with neutrophils and macrophage immune cells responding. As a result of the IL-6 concentration, the amplification of inflammation-related signals or transformation from an acute to a chronic inflammatory state occurs [17,18]. Targeting prostaglandin production by inhibiting COX-2, the rate-limiting enzyme, is one of the options to successfully treat inflammatory skin diseases [8,19].

*Waltheria indica* L., belonging to the Malvaceae family, is a traditional medicinal plant with anti-inflammatory properties used by indigenous populations in different regions of the world for the treatment of various pathological conditions [20,21]. The biologically active compounds proved to be present in all parts of *Waltheria indica* with reports supporting the use of roots, stems or leaves for treatment against swelling, cough, toothache, sore throat, rheumatism or complicated ailments such as asthma and inflammatory skin diseases [21,22,23,24,25]. Studies on the properties of Waltheria Indica showed that its extracts have multiple anti-inflammatory activities, often without attributing the effect to a single molecule. Extracts that exhibit biological activity include various chemical groups such as alkaloids, flavonoids, sterols, terpenes, anthraquinones or carbohydrates [21,26,27]. Hydroalcoholic extracts of the whole plant of *Waltheria indica*, for example, strongly inhibited edema at the second phase of carrageenan inflammation in rats [28]. Extracts from leaves generated with hydrophobic solvents such as petrol ether or methanol, but also extracts from leafy stems generated with hydrophilic solvents such as water, showed dose-related inhibition of acute and chronic inflammation in carrageenan-induced edema. The effect is presumed to involve the inhibition of histamine, serotonin, bradykinin, prostaglandin and cyclooxygenase (COX) products [29,30,31].

Studies that aimed at uncovering the anti-inflammatory molecules in Waltheria identified three flavonoids ((-)-epicatechin, quercetin and tiliroside) and two alkaloids (waltherione A and C) as potential active molecules [21,32,33]. The alkaloids waltherione A and C, obtained from the decoction of roots and aerial parts, were shown to inhibit nuclear factor (NF-κB) [34]. Quercetin and tiliroside showed a dose-dependent inhibition of the production of inflammatory mediators, including nitric oxide (NO), tumor necrosis factor (TNF-alpha), interleukin (IL)-12 and COX-2 [32,35,36]. Furthermore, it was suggested that the reason for the observed inhibition of lipoxidase-5 (5-LOX) and phospholipase A2 (PLA2) by the hydroalcoholic Waltheria extracts is due to (-)-epicatechin, which, in other studies, demonstrated COX-2 inhibitory properties [21,33,37,38]. It is important to mention that although these molecules exhibited efficacy in the performed assays, it was not evident from these studies whether their content in the Waltheria extracts is sufficient or different substances contribute to—or are responsible for—the observed activity. In addition to the substances isolated from *Waltheria indica*, other classes of compounds have been explored in various plants that exhibit significant COX-2 inhibitory activity, with flavonoids, alkaloids, terpenoids, saponins and fatty acids representing the most prominent ones that are presumably present but not yet identified in *Waltheria indica* [39].

Given the complexity of the plant extracts, the substances contributing to the anti-inflammatory effect of the extracts are regularly merely assumed and, in most cases, unknown or assigned to molecules whose content in the extract is insufficient or not known at all. Consequently, in our previous study we attempted to clarify to what extent a known anti-inflammatory molecule present in *Waltheria indica*, namely tiliroside, contributes to the COX-2 inhibition activity in the extracts. We demonstrated that tiliroside inhibits COX-2 activity in a concentration-dependent manner. However, the correlation between tiliroside content and COX-2 inhibition could not be confirmed for the extracts. Extracts with the highest tiliroside content did not exhibit the highest COX-2 inhibition; in contrast, the highest inhibitory activity was found in extracts with low tiliroside content. This led to the conclusion that in these extracts, other, more potent substances must be responsible for the activity [40].

A study identifying the substances responsible for the COX-2 inhibitory activity of *Waltheria indica* leaf extracts is yet not described. Therefore, the aim of this study was to identify and quantify the molecules responsible for the COX-2 inhibitory activity of the extracts using an activity-guided approach through extract fractionation by centrifugal partition chromatography (CPC). Ideally, it should be verified whether and to what extent the COX-2 inhibitory effect correlates with the content of these substances in the extracts. In addition, the different CPC fractions produced were analyzed for their secondary metabolite composition in terms of phenols, and triterpenoid and steroidal saponin content to ideally allow the generation of a prediction model to quantify the COX-2 inhibitory activity without biological testing.

The activity-guided approach led to the identification of three fatty acids, which were identified and reported for the first time in *Waltheria indica* leaf extracts. The investigations provide evidence that these fatty acids are responsible for up to 41% of the COX-2 inhibition observed in the respective *Waltheria indica* extract. Additional phytochemical analyses of the extract fractions revealed that substances from the group of steroidal saponins and triterpenoid saponins contribute to the COX-2 inhibitory properties of the extracts. The identification of substances contributing to COX-2 inhibition enabled the successful development of mathematical models to predict the COX-2 inhibition of *Waltheria indica* extracts, allowing the preparation of extracts with enhanced anti-inflammatory properties in future studies.

## 2. Results and Discussion

### 2.1. Centrifugal Partition Chromatography Fractionation

With the goal of obtaining fractions composed of different compounds of the *Waltheria indica* leaf extract, the separation was carried out using CPC coupled with a diode array detector (DAD). For this purpose, Waltheria leaves were extracted utilizing accelerated solvent extraction (ASE) at 70 °C with ethanol. To determine the appropriate solvent composition for the CPC, a systematic approach was adapted as described in the literature, using the Arizona liquid system (AZ) with the solvent composition N (AZ-N) as the starting point [41]. The method was performed in two steps, where step 1 was the elution step with a column volume of mobile phase and a run-time of 50 min. Step 2 was the extrusion step, replacing the entering mobile phase with the stationary phase. Outcomes with solvent compositions that had either formed an unstable system or presented no added value according to the AZ system were not listed. The yields of the individual dried fractions from separate CPC runs and the recovery rate of the injected extract amount are summarized in Table 1.

**Figure 1:**
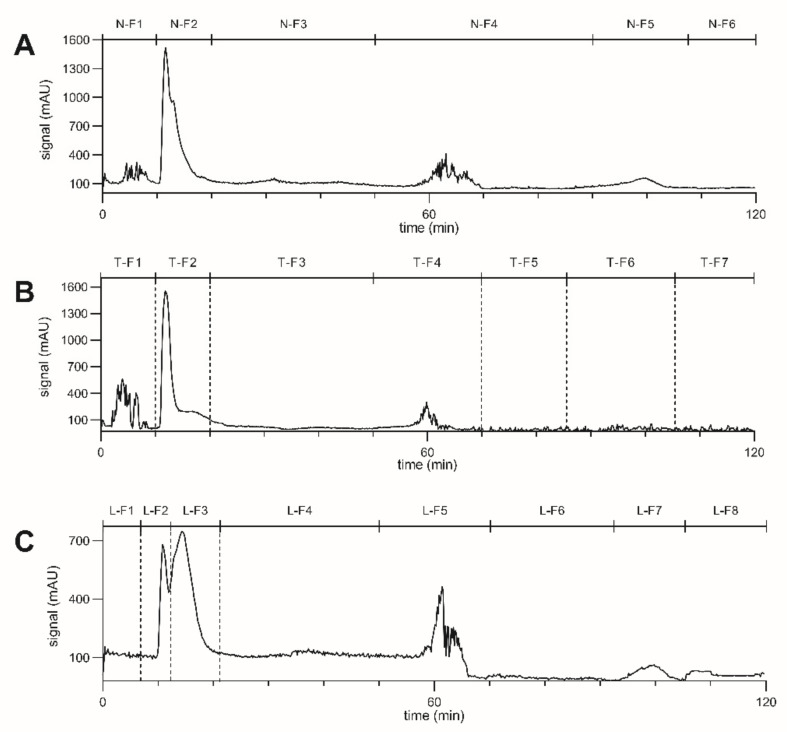
Chromatograms at 254 nm from CPC fractionations with *Waltheria indica* leaf extract and Arizona liquid system with (**A**) solvent composition N, (**B**) solvent composition T and (**C**) solvent composition L. The elution step lasted for 50 min initially, followed by an extrusion step. Fractions highlighted with dashed lines were further analyzed by UHPLC.

From the obtained yields (Table 1), it was evident that with AZ-N the extract was separated into two main fractions: N-F2 (55.3% yield) containing the more polar compounds and N-F5 (39.9% yield) containing the more nonpolar compounds. The observed signals at N-F1 and N-F4 were solvent turbulences (Figure 1A). For N-F1, this was due to sample injection, and for N-F4, this was due to the transition from the elution to the extrusion step. These effects were also observed for the AZ-T and AZ-L runs at the corresponding time periods (Figure 1B,C).

To separate the non-polar portion of the extract into additional fractions, CPC was performed with less polar solvent compositions than AZ-N, with AZ-T proving to be the appropriate choice. While the polar extract fraction was primarily found in fractions T-F2 (49.6% yield) and T-F3 (8.7% yield), the non-polar fraction could be divided into fractions T-F5 containing 13.2% of the extract and T-F6 with 27.5% (Table 1). Fractionation into T-F5 and T-F6 was primarily conducted on a visual basis, with T-F5 being yellow and T-F6 being green, as no UV signal was detectable with CPC-DAD (Figure 1B).

The use of the more polar solvent composition of the AZ-L system as compared to AZ-N allowed the separation of the polar extract fraction into two separate fractions: L-F2 with 31.5% yield and L-F3 with 25.0% yield (Figure 1C). Chromatograms of the CPC fractionations are shown in Figure 1. Fractions highlighted with dashed lines were selected for testing of their COX-2 inhibitory activity and further analyzed by UHPLC.

**Figure 2:**
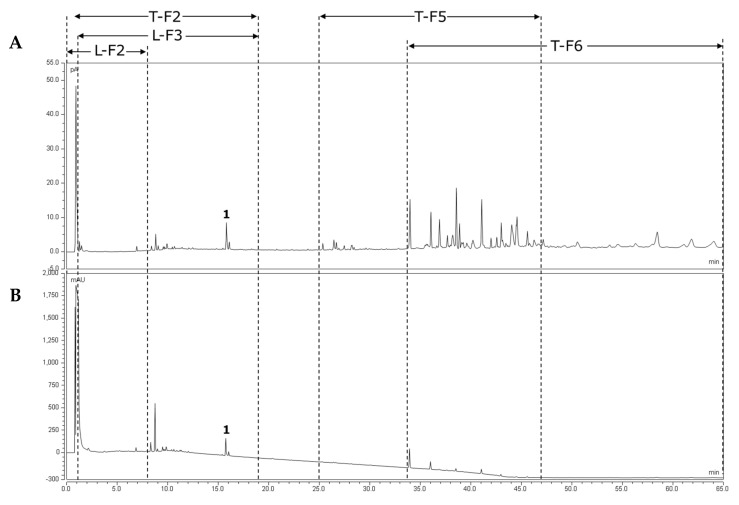
UHPLC chromatogram of *Waltheria indica* leaf extract E70 obtained at 70 °C with ethanol; the dashed lines show the observed compounds of the respective CPC fractions with (**A**) CAD and (**B**) DAD at 200 nm. The signal (**1**) at Rt = 15.81 min corresponds to that of tiliroside.

The Extract E70, the two nonpolar fractions T-F5 and T-F6, the polar fractions L-F2 and L-F3, and the fraction T-F2 were analyzed by UHPLC-CAD and -UV. For a better overview, the observed compounds of the CPC fractions are displayed in the chromatogram of the extract E70 (Figure 2). Substance (1) at Rt = 15.92 min is tiliroside (supplementary data), already identified in our previous study [40]. The chromatograms of the individual CPC fractions are summarized in Supplementary Materials (Figures S1–S5).

The fraction T-F2 contained substances up to Rt = 19 min, while the fraction L-F2 primarily combined the substances up to Rt = 8 min. The fraction L-F3 contained less polar compounds up to Rt = 19 min, including tiliroside, which was not present in L-F2. In contrast, the fractions T-F5 and T-F6 collected molecules after Rt = 25 min, with fraction T-F5 containing substances up to Rt = 47 min and T-F6 containing substances from Rt = 33.5 min. The molecules present in the first half of the overlap region of fractions T-F5 and T-F6 were more prominent in T-F5, whereas the molecules in the second half of the overlap region were more prominent in T-F6. With the aim of identifying the most active compounds, these five fractions were tested for their COX-2 inhibitory activity.

### 2.2. COX-2 Inhibitory Activity of CPC Fractions

**Figure 3:**
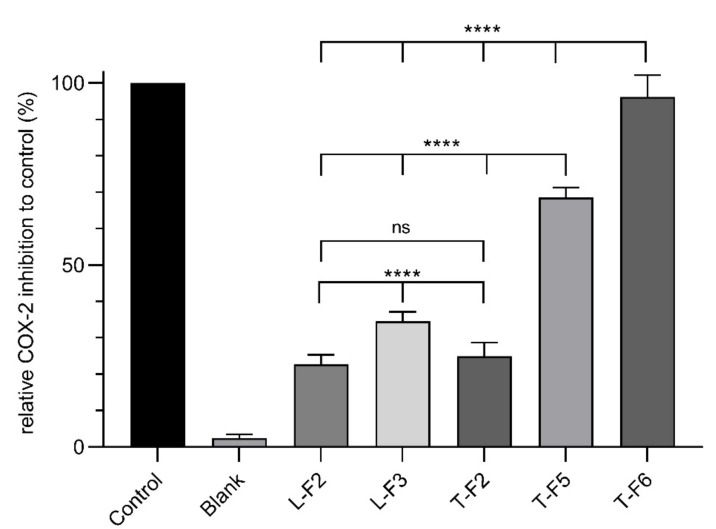
Relative COX-2 inhibition to control (%) of CPC fractions (20 µg/mL), blank (DMSO) and control set to 100% (0.5 µM celecoxib). Data points represent the mean value ± standard deviations of six samples. Statistically different expressions were calculated using one-way ANOVA. (****): *p* < 0.0001; (ns): not significant.

The results of the relative COX-2 inhibition for the CPC fractions are summarized in Figure 3. For comparison, the positive control was set to 100% and the samples were calculated accordingly.

The generated CPC fractions exhibited a COX-2 inhibitory effect. The polar fractions L-F2, L-F3 and T-F2 showed COX-2 inhibitory activity levels of 22.8%, 34.6% and 25.1%, respectively. Among these CPC fractions, L-F3 showed the highest activity. This could be due to a higher enriched tiliroside concentration, which was either not present (L-F2) or represented a smaller part (T-F2) in the other fractions.

The nonpolar fractions T-F5 and T-F6 exhibited a significantly higher COX-2 inhibitory activity with 68.7 and 96.3%, respectively. These results indicate that the nonpolar fractions comprised compounds which are more potent than those in the polar fractions, supporting the similar observations of a recent study [40]. At that time, it was found that extracts produced with more polar solvents showed a weaker COX-2 inhibitory activity than extracts produced with non-polar solvents. Considering that the T-F5 and T-F6 fractions exhibited the highest activity, these CPC fractions were selected to elucidate the structure of the molecules responsible for the COX-2 inhibitory activity.

### 2.3. Structural Elucidation of the Molecules in Fraction T-F5 and T-F6

**Figure 4:**
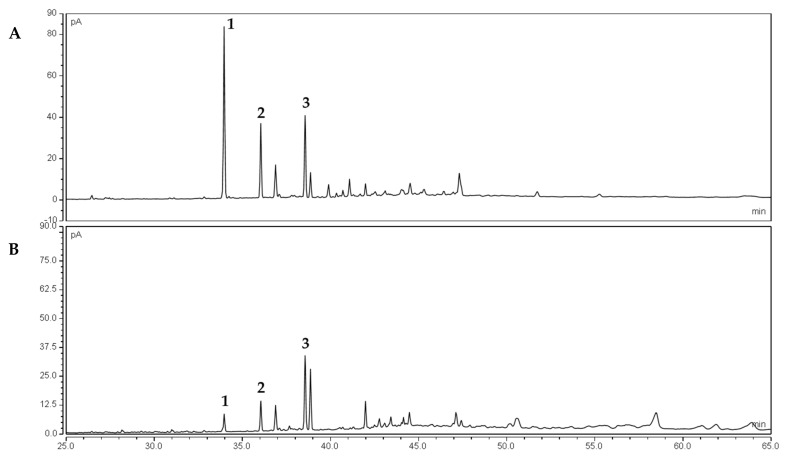
UHPLC-CAD chromatogram of the CPC fraction (**A**) T-F5 and (**B**) T-F6. The signal (1) at Rt = 33.91 min corresponds to alpha-linolenic acid, (2) that at Rt = 36.00 min corresponds to linoleic acid, and (3) that at Rt = 38.51 corresponds to oleic acid.

Three signals present in both the fraction T-F5 and T-F6 were identified by high resolution mass spectrometry (HPLC-MS/MS) with further verification by comparing fragmentation data with the respective internal standard (Figures S6–S12). The molecules identified and illustrated in Figure 4 are the fatty acids (FA) alpha-linolenic acid (1), linoleic acid (2), and oleic acid (3), which, to the best of our knowledge, were here identified and reported for the first time in *Waltheria indica* leaf extracts.

All three compounds have been reported to be COX-2 inhibitors, with alpha-linolenic acid (ALA) and linoleic acid (LA) being described as substantially more efficient than oleic acid (OA) [42,43]. The COX reaction produces prostaglandins by converting arachidonic acid (AA). However, this enzyme is capable of oxidizing other unsaturated fatty acids including alpha-linolenic acid and linoleic acid [44,45]. Structural determinations have revealed that, similarly to AA, these FA bind in elongated L-shaped conformations within COX-2, suggesting that their inhibitory activity originates in competing with arachidonic acid as substrates for COX-2 [43,46].

After having identified the three fatty acids, they were quantified in different *Waltheria indica* leaf extracts and in the CPC fractions T-F5 and T-F6.

### 2.4. Quantification of ALA, LA, OA in Extracts and CPC Fractions

**Figure 5:**
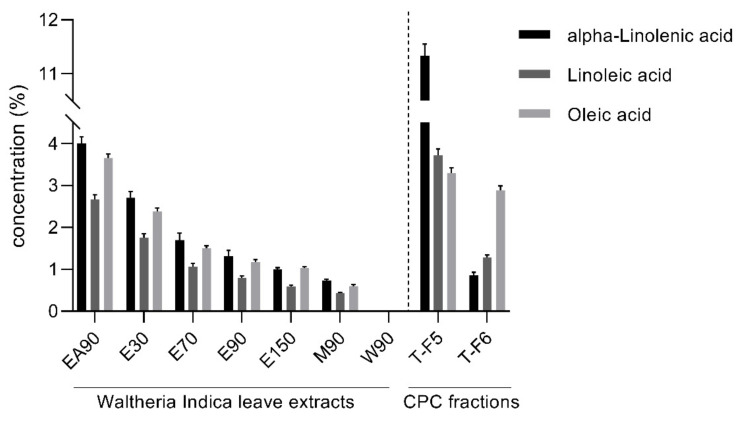
Concentration (%, *w*/*w*) of alpha-linolenic acid, linoleic acid and oleic acid in dried *Waltheria indica* leaf extracts obtained by varying extraction temperatures and solvents (left of dashed line) as well as CPC fractions T-F5 and T-F6 obtained from extract E70 (right of dashed line). Data points represent the mean value ± standard deviations of three samples. EA90, 90 °C ethyl acetate; E30–E70–E90–E150, 30–70–90–150 °C ethanol; M90, 90 °C methanol; W90, 90 °C water.

With the aim of investigating the influence of the extraction parameters on the extraction of ALA, LA and OA from Waltheria leaves, their concentrations were quantified in extracts obtained by varying the extraction temperatures and solvents. Furthermore, their content was determined in the CPC fractions T-F5 and T-F6 as it was evident from the chromatograms prepared previously that these FA were not present in fractions L-F2, LF-3 and T-F2. The quantification of the FA further made it possible to verify, in further steps, whether their amount was sufficient for the COX-2 inhibitory activity in the samples. The concentrations (%, *w*/*w*) of ALA, LA and OA in the respective dry extract and in dried CPC fractions are summarized in Figure 5, with the extracts sorted in decreasing order by their contents of fatty acids. In addition, the extracted amounts of ALA, LA and OA from 1 g of dry plant material (mg/g plant) were calculated following Equation (2) and are listed in Table 2.

The extract obtained with ethyl acetate as a solvent at 90 °C (EA90) showed, with 4.0% ALA, 2.7% LA and 3.7% OA, the highest concentration of all three fatty acids among all extracts. The second highest concentration of the three fatty acids was observed in the extracts obtained after extraction with ethanol (E30–E150). The results showed that the increase in the extraction temperature from 30 °C (E30) up to 150 °C (E150) caused a reduction in the fatty acids in the extract (Figure 5). In contrast, the total amount of ALA, LA and OA extracted from 1 g plant material with ethanol increased with higher extraction temperature (Table 2). It can be inferred that a higher temperature contributes to the disruption of the plant cell wall, which enables the solvent to extract more lipids, while simultaneously extracting larger amounts of additional substances, resulting in a reduced content of fatty acids in the total extract. Extract E30 contained 2.7% ALA, 1.8% LA and 2.4% OA, whereas 1.0% ALA, 0.6% LA and 1.0% OA were present in extract E150. The concentration of the fatty acids in the extracts produced at 70 °C (E70) and 90 °C (E90) were 1.7% ALA, 1.1% LA and 1.5% OA, and 1.3% ALA, 0.8% LA and 1.2% OA, respectively. After extraction with methanol at 90 °C, the extract (M90) contained 0.7% ALA, 0.4% LA and 0.6% OA, the second lowest content of the fatty acids in the prepared extracts.

The investigations of the CPC fractions demonstrated that fatty acids were significantly more efficient enriched in the fraction T-F5 compared to T-F6. The concentrations of ALA, LA and OA in the fraction T-F5 were 11.3%, 3.7% and 3.3%, respectively. In fraction T-F6, the concentration was significantly lower with 0.9% ALA, 1.3% LA and 2.9% OA.

Based on the presented data, it is evident that ALA is the most abundant fatty acid in Waltheria leaves, followed by OA and LA. The results also highlight the influence of the polarity of the solvents. Solvents such as ethyl acetate and ethanol favor the accumulation of fatty acids in the extract more than the accumulation of polar solvents, methanol and water (Figure 5).

In addition, the results demonstrated that the highest extract yield was obtained with the most polar solvents (water > methanol > ethanol > ethyl acetate) operating at 90 °C, whereas increasing the temperature for ethanol extraction resulted in a higher extract yield (E30–E150). As a result of the overall higher extract yield with ethanol (E90) and methanol (M90) over ethyl acetate (EA90), more fatty acids were extracted per 1 g of dry plant material with these solvents (Table 2). The reason for the lower content of fatty acids in these extracts can be attributed to the fact that they contained larger amounts of additional polar substances, resulting in a dilution of the fatty acids in the total extract.

After determining the content of the individual fatty acids in the extracts and CPC fractions, the next step was to investigate the effect of the pure fatty acids and the obtained extracts in terms of their COX-2 inhibitory activity.

### 2.5. COX-2 Inhibitory Activity of ALA, LA, OA and Extracts

**Figure 6:**
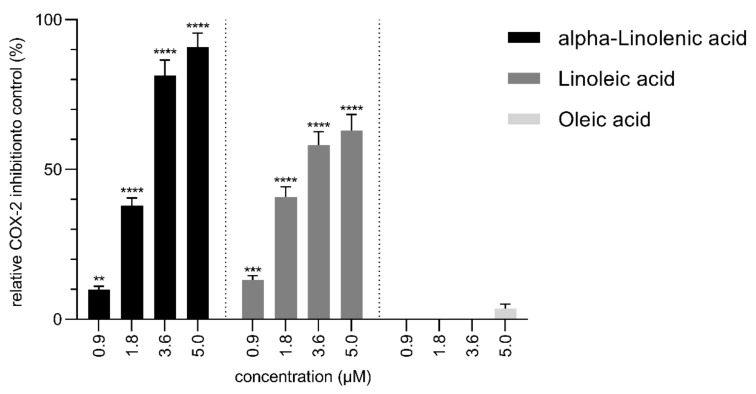
Relative COX-2 inhibition to control (%) of alpha-linolenic acid, linoleic acid and oleic acid with 0.5 µM celecoxib as control set to 100%. Data points represent the mean value ± standard deviations of six samples. Statistically different expressions were calculated using one-way ANOVA. (****): *p* < 0.0001; (***): *p* = 0.0001; (**): *p* = 0.0029 vs. blank.

**Figure 7:**
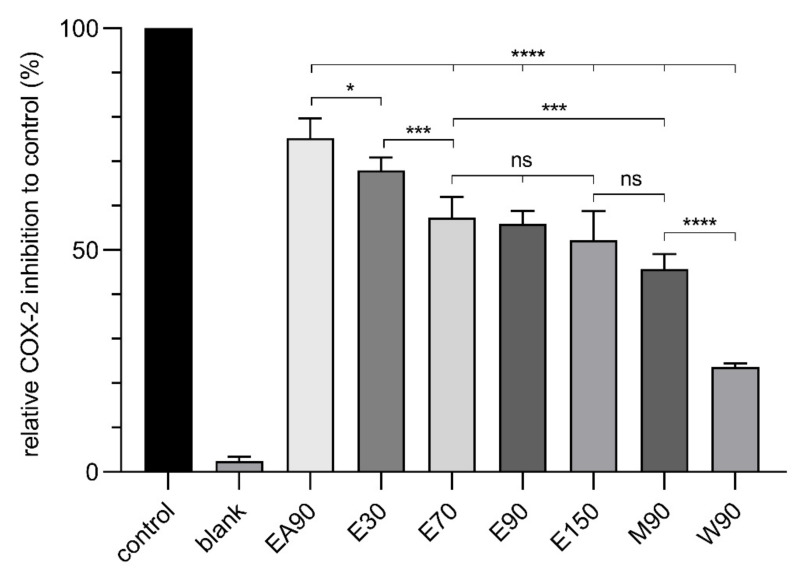
Relative COX-2 inhibition to control (%) of *Waltheria indica* extract solution (20 µg/mL), blank (DMSO) and control set to 100% (0.5 µM celecoxib). Data points represent the mean value ± standard deviations of six samples. Statistically different expressions were calculated using one-way ANOVA. (****): *p* < 0.0001; (***): *p* = 0.0005; (*): *p* = 0.0442; (ns): not significant.

The COX-2 inhibitory activity of pure alpha-linolenic acid, linoleic acid and oleic acid was studied in the concentration range from 0.9 to 5.0 µM, which includes the concentration levels observed in the extracts and shown in Figure 6. Similarly, the extracts were analyzed for their COX-2 inhibitory activity and displayed in Figure 7.

The COX-2 inhibition of ALA and LA increased in a dose-dependent manner starting at a concentration of 0.9 µM with 9.9% inhibition for ALA and 13.1% inhibition for LA and increasing up to 90.8% for 5.0 µM ALA and 63.0% for 5.0 µM LA. Oleic acid showed a measurable activity only at 5 µM, with 3.5% activity. The results are consistent with previous reports that ALA and LA possess COX-2 inhibitory properties, whereas OA shows activity only at very high concentrations [42,43].

The studied extracts were sorted in decreasing order according to their ALA and LA contents, with EA90 containing the highest and W90 the lowest amount; they are plotted against their respective COX-2 inhibition activity in Figure 7. Knowing that oleic acid does not make a significant contribution to COX-2 inhibition at the concentrations present in the extracts, the following results will focus primarily on ALA and LA.

The results indicate that the COX-2 inhibitory activity of the extracts declines with the decreasing of the fatty acids’ concentrations. The observed relative COX-2 inhibitory activity was 75.4% for EA90, 67.9% for E30, 57.5% for E70, 56.0% for E90, 52.5% for E150, 45.8% for M90 and 23.7% for W90.

The extract EA90, with the highest concentration of ALA and LA content (4.0% ALA, 2.7% LA), corresponds to a concentration of 3.1 µM pure ALA and 2.0 µM pure LA in the assay. Based on the results in Figure 6, inhibition in the 60–80% range would be expected, with 3.0 µM pure ALA alone matching the actual observed inhibition of 75.4%.

The observation that one fatty acid may be accountable for the complete COX-2 inhibition of the extract could not be made for the other extracts. Extract E30, for example, with ALA and LA concentrations in the assay corresponding to 2.0 µM ALA and 1.3 µM LA, would be expected to have an activity in the range between 30 and 40%. However, a COX-2 inhibitory activity of 67.8% was measured. The comparison of the results of E70 and E150 further indicated that the concentration of fatty acids in these extracts does not translate 1:1 to the scaling of COX-2 inhibition of the extracts. E70 contained 1.7-fold higher ALA content and 1.8-fold higher LA content relative to E150, whereas the COX-2 inhibition of these extracts did not reflect this increase.

Based on these data, no definitive statement could be made regarding the exact contribution of the fatty acids to the COX-2 inhibition of the extracts. Therefore, the next step was to test for the presence of fatty acid mixtures (FAM) containing ratios of ALA, LA and OA in the respective extracts and examine their COX-2 inhibition.

### 2.6. COX-2 Inhibitory Activity of Fatty Acid Mixtures (FAM)

To investigate the accurate contribution of the fatty acids to the observed COX-2 inhibitory activity of the extracts, the formulated FAM of ALA, LA and OA, at a concentration and ratio identical to the corresponding extract, were prepared and analyzed for their COX-2 inhibitory activity. In addition, the contribution of the fatty acids to the total COX-2 inhibitory activity of the respective extract was calculated using Equation (6) and the results are summarized in Table 3 with the extracts sorted in decreasing order according to their fatty acid contents, with EA90 containing the highest and W90 the lowest amount.

The results of the FAM indicated a similar trend to the one obtained from the corresponding extracts, showing a decline in activity as the concentration of fatty acids decreased. The contribution of fatty acids, when present in the extract, to COX-2 inhibition of the extract ranged from a minimum of 31.3% (M90) up to 41.6% (E70). These findings provide clear evidence that the identified fatty acids, at their observed concentrations in the extracts, contribute substantially to the COX-2 inhibition observed with the respective *Waltheria indica* extracts and enable the preparation of extracts with enhanced anti-inflammatory properties by choosing extraction parameters that result in a higher ALA or LA presence in the extract.

To further characterize the compounds involved in the COX-2 inhibitory activity, the phytochemical compositions of the CPC fractions were further investigated.

Before that, it was assessed whether antioxidants play a role in COX-2 inhibition. The results showed no relationship between the COX-2 inhibitory activity of the samples and their corresponding antioxidant properties. The details are summarized in the Supplementary Material section for the purpose of completeness (Figure S13).

### 2.7. Phytochemical Composition of the CPC Fractions

The phytochemical composition of the CPC fractions was examined with the aim of characterizing further compounds with COX-2 inhibitory activity, given that they differ significantly in activity and composition. The quantifications of total phenols, and triterpenoid and steroidal saponins in each CPC fraction are summarized in Table 4. For a more complete overview, their ALA and LA contents, as well as the associated COX-2 inhibition levels, are listed.

The highest phenolic contents were observed in the polar fractions L-F2, L-F3 and T-F2 with 288.3, 220.1 and 256.8 mg/g, respectively, which were significantly higher compared to the nonpolar fractions T-F5 and T-F6 with 24.2 and 21.0 mg/g. In contrast, the polar fractions contained significantly lower levels of triterpenoid saponins with 49.2, 77.5 and 67.5 mg/g for L-F2, L-F3 and T-F2, respectively, compared to T-F5 with 325.8 mg/g and T-F6 with 188.5 mg/g (Table 4). For steroidal saponins, fraction T-F6 contained the highest amount with 107.4 mg/g, followed by T-F5 with 30.8 mg/g, L-F3 with 26.5 mg/g, L-F2 with 19.3 mg/g and T-F2 with 18.8 mg/g (Table 4).

When considering the total phenol content in the polar fractions L-F2, L-F3 and T-F2 and their COX-2 inhibition, a correlation was not evident as L-F2, with the highest phenol content, had a lower COX-2 inhibitory activity compared to L-F3 with the lowest phenol content but the highest COX-2 inhibition. In the case of the triterpenoid- and steroidal saponin content, one could deduce that COX-2 inhibition increases with its increasing content in these fractions.

In the case of nonpolar fractions T-F5 and T-F6, the results revealed that although T-F6 contained lower content of triterpenoid saponins, ALA and LA, compared to T-F5 the observed COX-2 inhibitory activity was significantly higher. This can be explained by the steroidal saponin content, which was 3.5 times higher in fraction T-F6, indicating that steroidal saponins in this case contributed predominantly to the COX-2 inhibitory activity.

Based on these data, it can be concluded that the steroidal and triterpenoid saponins, and ALA and LA, are among the prominent COX-2 inhibitory compounds in *Waltheria indica* extracts. With the aim of predicting the COX-2 inhibitory activity of the extracts, the next step was to attempt the development of mathematical models based on the composition and COX-2 inhibitory activity of the extracts studied.

### 2.8. COX-2 Inhibition Prediction Models

To predict the COX-2 inhibitory properties of different *Waltheria indica* extracts, two mathematical models (model A and B) were generated, considering steroidal and triterpenoid saponins as well as ALA and LA as positive contributors to the COX-2 inhibition. The phytochemical composition of the investigated extracts and their COX-2 inhibition activity served as the data basis (Table S1). Tiliroside, OA and phenols were not taken into consideration since experimental work indicated that they do not influence the COX-2 inhibitory activity at the concentration levels present in the extracts.

Model A was generated by considering, as a predictor variable for COX-2 inhibition activity, the weighted sum of the concentrations of the contributors in the extract. For each contributor, the slope of the linear regression line between the concentration of the contributor and COX-2 inhibition in the extracts served as the weighting factor. For ALA and LA, we observed a second-order polynomial relationship between their concentration and COX-2 inhibition activity. Thus, a linearization was applied to both variables, in this case a square root transformation, to obtain the slope of the linear regression line. The obtained weighting factors for the individual concentrations were 0.27 for triterpenoid saponins, 0.50 for steroidal saponins, 8.23 for the square root of ALA and 10.03 for the square root of LA. A linear regression line was then fit between the weighted sum of contributors (x-variable) calculated using Equation (7) and COX-2 inhibition activity (y-variable), leading to the equation y = 0.257x + 20.911.

Model B was generated using partial least squares (PLS) regression, which is a linear multivariate regression that is useful for analyzing data with many variables and few observations (as is in the present case: four variables with seven observations). The PLS regression considered as predictors (x-variables) the concentration of each positive contributor and the COX-2 inhibitory activity as a response variable (y-variable). Similar to Model A, ALA and LA concentration values were first square-root transformed to improve their linear relationship with COX-2 inhibitory activity. The obtained PLS model can be represented by the linear equation y = 0.0693a + 0.1296b + 2.1269c + 2.5925d + 20.9035.

**Figure 8:**
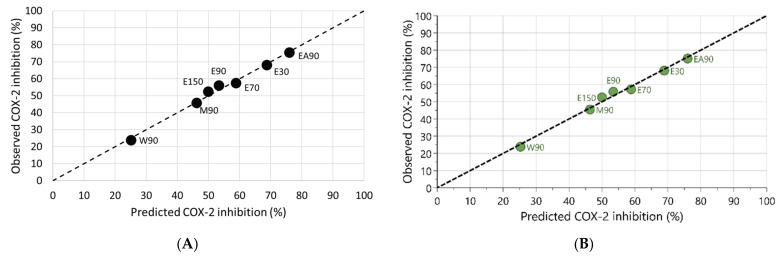
Graphs representing (**A**) model A linearity plot of observed versus predicted COX-2 inhibition of *Waltheria indica* extracts (RMSE = 1.6%) based on the obtained linear fit equation y = 0.257x + 20.911 generated by the weighted sum of the contributors versus COX-2 inhibitory activity and (**B**) model B linearity plot of observed versus predicted COX-2 inhibition (RMSE = 1.6%) based on the generated linear fit equation y = 0.0693a + 0.1269b + 2.1269c + 2.5925d + 20.9035 by PLS regression. Dashed lines represent the line of identity (y = x). y = predicted COX-2 inhibition (%); x = weighted sum concentration of the contributors; a = triterpenoid saponin concentration; b = steroidal saponin concentration; c = square root concentration of ALA; d = square root concentration of LA.

Both models were used to generate a linearity plot, i.e., the scatter plot showing the relationship between the observed versus predicted COX-2 inhibitory activity values for the extracts, and to calculate the root mean square error (RMSE). The obtained linearity plots are depicted in Figure 8.

The results demonstrate that the approach of using the weighted sum of the concentrations in model A led to similar results as the application of a multivariate regression method available in a commercial software carried out with method B. Applying the linear fit equation from model A or model B results in a similarly good fit with a RMSE = 1.6% COX-2 inhibition activity demonstrating a high correspondence between predicted versus observed values.

In both models, the data show that the coefficients of the contributors increase in the order LA > ALA > steroidal saponins > triterpenoid saponins, indicating that their contribution to COX-2 inhibitory activity in *Waltheria indica* extracts follows this order. It is also important to note that while these models provide good prediction results for *Waltheria indica* extracts, they are not necessarily applicable to CPC fractions or to pure substances. As it is important not to extrapolate, the concentration of the phytochemicals in the test samples should be within the concentration ranges of the extracts used for the models. While the individual fractions are simplified systems suitable for the identification of positive contributors to the COX-2 inhibitory activity, extracts are much more complex systems with a greater number of interactions between the individual components having an influence on the potential biological activity. This becomes obvious when looking at the single substances without any interactions with other compounds in the extracts, where ALA showed a stronger contribution overall compared to LA (Figure 6). In the case of the CPC fractions where the concentrations of several phytochemicals were higher compared to their concentration in extracts, the data suggest that the steroidal saponins contribute the most (Table 4). In the event of the future discovery of additional substances that contribute positively to COX-2 inhibition and are present in sufficient concentrations in the extract, the mathematical models established here should be revisited and can be adopted accordingly.

Based on the proposed mathematical models, a more targeted development of extraction procedures is possible in order to obtain *Waltheria indica* extracts with improved anti-inflammatory properties. Furthermore, the transfer of the approach presented in this work to the prediction of other biological endpoints would be of great interest and should be considered in future studies.

## 3. Materials and Methods

### 3.1. Chemicals

Gallic acid, oleanolic acid, diosgenin, alpha-linolenic acid, linoleic acid and oleic acid (as reference standards) and Folin–Ciocalteu reagent, vanillin, perchloric acid, sulfuric acid, dimethyl sulfoxide were purchased from Sigma-Aldrich (St. Louis, MO, USA). Sodium carbonate, acetic acid, hydrochloric acid and anisaldehyde were obtained from Merck KGaA (Darmstadt, Germany). All other solvents and chemicals used were of analytical or HPLC grade. The working solutions were prepared immediately prior to measurement.

### 3.2. Plant Material

*Waltheria indica* L. plant was collected from Wageningen University & Research (Wageningen, Gelderland, The Netherlands) and confirmed as *Waltheria indica* L. by Eurofins Genomics Europe Applied Genomics GmbH. Leaf samples were dried at a constant temperature of 40 °C for 48 h (Vacutherm, Thermo Scientific) and grounded into a fine powder before extraction.

### 3.3. Plant Extract Preparation

Plant extraction was performed by accelerated solvent extraction Dionex ASE 350 (Thermo Scientific, Waltham, MA, USA). For all extractions, 100 mL containers were charged with 10–12.5 g plant material mixed with diatomaceous earth (60-033854, Thermo Scientific, Waltham, MA, USA) as a neutral matrix to assure a 1:20 plant/solvent ratio. The ASE extraction was performed with 4 cycles in total, each cycle having a 6 min static time and a 160 s purge time at 1460 psi static pressure. The extraction temperature was 90 °C for all solvents and additionally 30, 70 and 150 °C for ethanol extraction. The obtained extracts were filtered through a 0.22 μm membrane (HPF Millex^®^, Merck Millipore, Burlington, MA, USA) and evaporated under vacuum at 60 °C. The extract yield (mg/g dry plant) was calculated using Equation (1) and the fatty acid yield extracted from 1 g plant material (mg/g dry plant) was calculated using Equation (2).

Yield extract = weight of dry extract/weight of dried plant material (1)

Yield fatty acid = concentration fatty acid in extract × yield extract(2)

### 3.4. Centrifugal Partition Chromatography

CPC separations were performed with a CPC-1000 (Gilson, Middleton, WI, USA) using a two-phase solvent system of the Arizona solvent family composed of heptane, ethyl acetate, methanol and water. Fractionation of the crude extract started with the solvent composition AZ-N (1:1:1:1, *v*/*v*). Subsequently, more polar solvent compositions (AZ-H (1:3:1:3, *v*/*v*), AZ-K (1:1:1:2, *v*/*v*) and AZ-L (2:3:2:3, *v*/*v*)) and non-polar solvent compositions (AZ-T (3:1:3:1, *v*/*v*), AZ-R (2:1:2:1, *v*/*v*) and AZ-U (4:1:4:1, *v*/*v*)) were carried out. The fractionation of the extract was performed in the descending mode. The column was first filled with the stationary phase, then the apparatus was rotated at 1500 rpm and the CPC column was equilibrated with the mobile phase at a flow rate of 20 mL/min. After having reached the hydrodynamic equilibrium, a sample solution (50 mL, 3 mg/mL) was injected into the column. The separation was performed at a flow rate of 20 mL/min and monitored with a DAD detector at 254 nm. The fractions were collected in 25 mL test tubes and evaporated under vacuum at 40 °C. The strategy for the combination of the fractions was based on the UV signal for the polar part of the plant. During the investigations, it became apparent that a number of compounds were not UV active. In particular, the fractionation of the non-polar part of the extract was partly realized on a visual basis, as the fractions showed color differences to some extent, with no detectable UV signal.

The CPC fraction yield (%, *w*/*w*) was calculated using Equation (3). The calculation of the non-polar part was primarily conducted on a visual basis, with fraction T-F5 being yellow and T-F6 being green, as no UV signal was detectable with CPC-DAD. The obtained fractions were dissolved in DMSO for further analysis (2 mg/mL).

Yield CPC fraction = weight of dry fraction/injected amount of dry extract × 100(3)

### 3.5. Identification of Fatty Acids and UHPLC Analysis

The identification of alpha-linolenic acid, linoleic acid and oleic acid in the CPC fraction T-F5 was achieved using the Vanquish HPLC system coupled with a mass spectrometer (Q Exactive™ Hybrid Quadrupole-Orbitrap™, Thermo Scientific, Waltham, MA, USA) with H-ESI interface and negative ionization with 30 °C column temperature, 0.4 mL/min flow speed and 1 µL injection volume.

The separation was accomplished using a C-18 column (ThermoFisher™ Hypersil Gold™ aQ, 150 × 2.1 mm, 1.9 μm, Thermo Scientific, Waltham, MA, USA) and gradient elution with solvent A (water/formic acid 99.9/0.1 (*v*/*v*)) and B (acetonitrile/formic acid 99.9/0.1 (*v*/*v*)). The gradient started at 5% (*v*/*v*) B, was held constant for 1 min, ramped up to 50% over 20 min, ramped up to 98% over 20 min, and held constant for 25 min.

The HPLC-MS/MS chromatograms are summarized in the Supplementary Material section. The HPLC profile of the sample T-F5 showed three intense UV peaks at Rt = 33.91 min with a single charge state of MH-277.2 g/mol, Rt = 36.00 min with MH-279.2 g/mol and Rt = 38.51 min with MH-281.2 g/mol. High resolution mass spectrometer fragmentation data confirmed the identification of alpha-linolenic acid, linoleic acid and oleic acid within the CPC fraction T-F5 (Figures S6–S12).

The analysis of the CPC factions and extract samples as well as the quantification of alpha-linolenic acid, linoleic acid and oleic acid were carried out on a UHPLC (UltiMate 3000, Thermo Scientific, Waltham, MA, USA) coupled with a CAD (Corona™ Veo™ RS, Thermo Scientific, Waltham, MA, USA) and a DAD (VANQUISH™ DAD HL, Thermo Scientific, Waltham, MA, USA). CAD detection was performed at 35 °C evaporation temperature and UV detection at 200 nm with 30 °C column temperature, 0.4 mL/min flow speed and 3 µL injection volume. The separation was accomplished with the identical column and method as used for the identification of the fatty acids by HPLC-MS/MS. The UHPLC-CAD chromatograms of the CPC fractions are summarized in the Supplementary Data (Figures S1–S5).

ALA, LA and OA concentrations present in the extracts and CPC fractions were calculated from a five data point calibration curve with the respective reference standard (0.010–0.140 mg/mL). The results were expressed in %, *w*/*w*.

### 3.6. COX-2 Inhibitory Activity

The ability of the CPC fractions and *Waltheria indica* extracts to inhibit COX-2 was determined using the fluorometric COX-2 specific inhibitor screening kit (BioVision, Zurich, Switzerland). The experimental protocol was followed according to the user manual. Fluorescence values (Ex/Em = 535/587 nm) of the samples were kinetically measured using a Tecan-Spark multimode microplate reader (Spark 20M, TECAN, Männedorf, Switzerland) at 25 °C for 10 min. Two appropriate points (T1 and T2) in the linear range of the plot were chosen, and the corresponding fluorescence values (RFU1 and RFU2) were obtained. The enzymatic assay was applied in the concentration range of 0.9–5 µM for the ALA, LA and OA reference standards and 20 µg/mL for the extracts and CPC fractions dissolved in dimethyl sulfoxide (DMSO). Each sample was analyzed six-fold; celecoxib (0.5 µM) was used as a positive control, while DMSO was used as a blank. The slope for all samples (S), including enzyme control (EC), was calculated by dividing the ∆RFU (RFU2–RFU1) values by the time ∆T (T2–T1). Subsequently, the percentage of relative COX-2 inhibition (RI) of the samples was first calculated using Equation (4):

% relative COX-2 inhibition = (Slope of EC − Slope of S)/Slope of EC × 100(4)

For a more accurate comparison of the results with subsequent experiments, the positive control was set to 100% and the percentage of relative COX-2 inhibition to the positive control was calculated according to Equation (5):

% relative COX-2 inhibition to positive control = RI sample/RI positive control × 100 (5)

The contribution, in percentage, of the fatty acid mixtures to the total COX-2 inhibitory activity of the respective extract was calculated using Equation (6).

Contribution = COX-2 inhibition FAM/COX-2 inhibition extract × 100 (6)

### 3.7. Quantification of Phytochemicals

#### 3.7.1. Phenol Quantification

For a reliable correlation between concentration of phenolic compounds and their activity, the total phenol content was determined using the Folin–Ciocalteu method with slight modifications [47]. The test solutions (1.0 mL, 1 mg/mL) were mixed with the Folin–Ciocalteu reagent (10 mL, previously diluted in water 1:10, *v*/*v*) and sodium carbonate (Na_2_CO_3_; 8.0 mL, 75 g/L). The tubes containing the solutions were vortexed for 15 s and incubated for 120 min at room temperature for color development. The absorbance was measured at 765 nm against a blank (methanol) using a Varian Cary 60 UV–VIS spectrophotometer (Agilent, Santa Clara, CA, USA). All assays were performed in triplicate. Gallic acid (0.0100–0.150 mg/mL) was used for calibration and the results were expressed as mg of gallic acid equivalents per gram of dry extract (mg GAE/g).

#### 3.7.2. Triterpenoid Saponin Quantification

The triterpenoid content was determined based on the vanillin-perchloric acid method with slight modifications [48]. The test solution (200 µL, 1 mg/mL), in a 10 mL tube, was heated to evaporate the solvent and the solid was reconstituted in a vanillin-glacial acetic acid solution (300 µL, 5% *w*/*v*) and in perchloric acid (1.0 mL, 70%). The sealed samples were heated for 45 min at 60 °C and afterwards cooled in an ice-water bath followed by the addition of glacial acetic acid (4.5 mL). The absorbance of the sample solutions was measured at 540 nm against a blank using a Varian Cary 60 UV–VIS spectrophotometer (Agilent, Santa Clara, CA, USA). The blank was treated identically with the exception that no vanillin was used. Oleanolic acid (0.0090–0.4000 mg/mL in methanol) was used for calibration and the results were expressed as mg of oleanolic acid equivalents per gram of dry extract (mg OAE/g).

#### 3.7.3. Steroidal Saponin Quantification

The spectrophotometric quantification of the steroidal saponin content was based on the quantification of steroidal sapogenins method with minor modifications in which stable and reproducible results with several standards and without interference from sugars, sterols, fatty acid and vegetable oil were reported [49,50,51].

After the test solution (200 µL, 1 mg/mL), in a 10 mL tube, was heated to evaporate the solvent, the precipitate was dissolved in ethyl acetate (2.0 mL) and mixed with anisaldehyde/ethyl acetate solution (1.0 mL, 0.5%, *v*/*v*) and sulfuric acid (1.0 mL, previously diluted in ethyl acetate 1:1, *v*/*v*). The sealed samples were further incubated for 20 min at 60 °C and cooled afterwards in an ice-water bath followed by the addition of demineralized water (0.5 mL). The samples were incubated for 30 min before the absorbance of the sample solutions was measured at 430 nm against a blank using a UV–VIS spectrophotometer (Varian Cary 60, Agilent, Santa Clara, CA, USA). The blank was treated identically with the exception that no anisaldehyde was used. Diosgenin (0.0125–0.2000 mg/mL in methanol) was used for calibration and the results were expressed as mg of diosgenin equivalents per gram of dry extract (mg DE/g).

### 3.8. COX-2 Inhibition Prediction Models

Two mathematical models (model A and B) were generated to predict the COX-2 inhibitory properties of different *Waltheria indica* extracts, considering steroidal saponins (S) and triterpenoid saponins (T) as well as ALA and LA as positive contributors to the COX-2 inhibition. The phytochemical composition of the investigated extracts and their COX-2 inhibition activity served as the data basis.

Model A was generated by considering, as a predictor variable for COX-2 inhibition activity, the weighted sum of the concentrations of the contributors (*S_w_*) in the extract. The weighting factor for each contributor i (Wi) was taken as the slope of the linear regression line between the concentration of the contributor (Ci) and the COX-2 inhibitory activity of the extracts. For ALA and LA, a second-order polynomial relationship between their concentration and COX-2 inhibition activity was observed. To obtain the slope of the linear regression line, and thus, the corresponding Wi values, a linearization was applied to both variables—in this case, a square root transformation. The weighted sum (*S_w_*) in model A of the concentrations of the contributors in the extract was calculated following Equation (7):

(7) $$S_{W}=W_{S}\times C_{S}+W_{T}\times C_{T}+W_{ALA}\times \sqrt[{2}]{C_{ALA}}+W_{LA}\times \sqrt[{2}]{C_{LA}}$$

The weighted sum (x-variable) of each extract was plotted versus the respective COX-2 inhibitory activity (y-variable) to obtain the linear regression line. The obtained Wi was 0.27 for triterpenoid saponins, 0.50 for steroidal saponins, 8.23 for the square root of ALA and 10.03 for the square root of LA. The so-obtained equation y = 0.257x + 20.911 was used to generate the predicted versus observed COX-2 inhibitory activity linearity plot and to calculate the root mean square error (RMSE)

Model B was generated using PLS regression, which is a linear multivariate regression, computed with SICMCA^®^ (version 17.0), a multivariate data analysis software application (Sartorius AG, Goettingen, Germany). The model used, as predictor variables (x-variables), the concentration of the four positive contributors (considering the square root values for ALA and LA), and the COX-2 inhibitory activity was used as the dependent variable (y-variable). Before model fitting, variables were mean centered and scaled to unit variance. Model optimization was based on internal validation by considering leave-one-sample-out cross-validation (using a 95% confidence level). Based on the latter, the final PLS model dimensionality (i.e., the number of significant PLS components) was one, and all the predictor variables were found to be significant to model the response of interest (and thus, were included in the PLS model).

### 3.9. Statistical Analysis

All results are expressed as means ± standard deviation (SD). Determinations of phytochemical and fatty acid concentrations were performed in triplicate and the determination of COX-2 inhibition was performed with n = 6. Statistical data processing was carried out by one-way analysis of variance (ANOVA) to assess the statistical significance of the observed differences. The statistical analysis was carried out using GraphPad Prism 9.1.2 (GraphPad Software, San Diego, CA, USA). *p* < 0.05 was considered statistically significant.

## 4. Conclusions

In this study, we showed that the activity-guided approach led to the identification of alpha-linolenic acid, linoleic acid and oleic acid, which were identified and reported for the first time in *Waltheria indica* leaf extracts. The study revealed that they are responsible for up to 41% of the COX-2 inhibition observed in the respective *Waltheria indica* extract, with ALA and LA proving to be the major contributors, of the three fatty acids, to COX-2 inhibition. Through additional phytochemical analyses of the extract fractions, substances from the group of steroidal saponins and triterpenoid saponins were also identified as positive contributors to the COX-2 inhibitory activity. The identification of substances contributing to COX-2 inhibition enabled the successful development of mathematical models to predict the COX-2 inhibition of *Waltheria indica* extracts. Based on the mathematical models, it can be inferred that the contribution of the different substances in the *Waltheria indica* extracts follows the order linoleic acid > alpha linolenic acid > steroid saponins > triterpenoid saponins. These findings enable a more targeted development of extraction processes to obtain *Waltheria indica* extracts with superior anti-inflammatory properties.

## Figures and Tables

**Table 1:** Yields (%, *w*/*w*) of the individual fractions (F1–F8) and the recovery rate from CPC runs with Arizona liquid system and the medium polar solvent composition N (AZ-N), non-polar solvent composition T (AZ-T) and polar solvent composition L (AZ-L); n.a., not available. Data points represent the mean value ± standard deviations of three samples.

CPC Fraction	F1	F2	F3	F4	F5	F6	F7	F8	Recovery (%)
AZ-N yield (%)	0	55.3 ± 2.4	2.8 ± 0.1	1.5 ± 0.1	39.9 ± 2.0	0	n.a.	n.a.	99.3 ± 4.2
AZ-T yield (%)	0	49.6 ± 2.4	8.7 ± 1.2	0.8 ± 0.2	13.2 ± 1.4	27.5 ± 1.8	0	n.a.	99.7 ± 3.8
AZ-L yield (%)	0	31.5 ± 1.2	25.0 ± 1.6	1.9 ± 0.2	0	2.1 ± 0.2	38.8 ± 1.4	0	99.2 ± 3.6

**Table 2:** Summary of extract yield (mg/g plant) and fatty acid content (mg/g plant) of *Waltheria indica* leaf extracts obtained with varying extraction temperatures and solvents. EA90, 90 °C ethyl acetate; E30–E70–E90–E150, 30–70–90–150 °C ethanol; M90, 90 °C methanol; W90, 90 °C water; n.d., not detectable. Values are means ± standard deviations of triplicate measurements.

Sample	EA90	E30	E70	E90	E150	M90	W90
Yield extract (mg/g plant)	29.4	15.5	74.0	108.9	170.0	210.1	225.4
ALA (mg/g plant)	1.18 ± 0.04	0.42 ± 0.02	1.26 ± 0.12	1.43 ± 0.16	1.70 ± 0.07	1.53 ± 0.09	n.d.
LA (mg/g plant)	0.78 ± 0.03	0.27 ± 0.01	0.79 ± 0.05	0.87 ± 0.04	1.00 ± 0.06	0.90 ± 0.04	n.d.
OA (mg/g plant)	1.07 ± 0.03	0.37 ± 0.01	1.11 ± 0.05	1.29 ± 0.06	1.75 ± 0.04	1.26 ± 0.08	n.d.

**Table 3:** Relative COX-2 inhibition to control (%) of *Waltheria indica* extract solution (20 µg/mL) and fatty acid mixture solution (20 µg/mL) with 0.5 µM celecoxib as control set to 100%. Data points represent the mean value ± standard deviations of six samples.

	EA90	E30	E70	E90	E150	M90	W90
Extract COX-2 inhibition (%)	75.4 ± 4.0	67.9 ± 2.7	57.5 ± 4.3	56.0 ± 2.6	52.5 ± 5.9	45.8 ± 3.1	23.7 ± 0.7
Corresponding FAM COX-2 inhibition (%)	27.6 ± 1.1	26.2 ± 1.5	23.9 ± 2.1	21.7 ± 1.9	18.5 ± 0.7	14.3 ± 0.9	0.6 ± 0.9
Contribution of FAM to COX-2 inhibition (%)	36.6 ± 2.5	38.6 ± 2.4	41.6 ± 3.1	38.8 ± 2.7	35.2 ± 3.2	31.3 ± 2.5	0

**Table 4:** Phytochemical composition in mg/g dry sample and relative COX-2 inhibition to control (%) of the *Waltheria indica* leaf extract fractions. Data points represent the mean value ± standard deviations of three samples. GAE = Gallic acid equivalent; OAE = Oleanolic acid equivalent; DE = Diosgenin equivalent; n.d., not detected.

CPC Fraction	L-F2	L-F3	T-F2	T-F5	T-F6
Total phenols (mg GAE/g)	288.3 ± 4.1	220.1 ± 4.2	256.8 ± 3.1	24.2 ± 2.1	21.0 ± 1.7
Triterpenoid saponin (mg OAE/g)	49.2 ± 1.9	77.5 ± 2.1	67.5 ± 4.1	325.8 ± 4.8	188.5 ± 3.4
Steroidal saponin (mg DE/g)	19.3 ± 1.1	26.5 ± 0.9	18.8 ± 1.3	30.8 ± 1.5	107.4 ± 4.1
Alpha-linolenic acid (mg/g)	n.d.	n.d.	n.d.	113 ± 2.1	8.6 ± 0.7
Linoleic acid (mg/g)	n.d.	n.d.	n.d.	37 ± 1.5	12.9 ± 0.5
COX-2 inhibition (%)	22.8 ± 2.5	34.6 ± 2.4	25.1 ± 3.5	68.7 ± 2.5	96.2 ± 5.5

## Data Availability

Data are available from the authors upon reasonable request.

## Supplementary Materials

The following are available online, Figure S1: UHPLC chromatogram of CPC fraction L-F2 with (A) CAD and (B) DAD at 200 nm. Figure S2: UHPLC chromatogram of CPC fraction L-F3 with (A) CAD and (B) DAD at 200 nm. Figure S3: UHPLC chromatogram of CPC fraction T-F2 with (A) CAD and (B) DAD at 200 nm. Figure S4: UHPLC chromatogram of CPC fraction T-F5 with (A) CAD and (B) DAD at 200 nm. Figure S5: UHPLC chromatogram of CPC fraction T-F6 with (A) CAD and (B) DAD at 200 nm. Figure S6: UHPLC-UV chromatogram at 200 nm of (A) CPC fraction T-F5 (2 mg/mL) and (B) alpha-linolenic acid reference standard (0.05 mg/mL), (C) linoleic acid (0.05 mg/mL), (D) oleic acid (0.05 mg/mL). Figure S7: MS data of (A) peak at Rt = 33.91 with a single charge state of MH-277.2 g/mol and (B) alpha-linolenic acid reference standard. Figure S8: Fragments (A) of 277.2 g/mol signal in fraction T-F5 and (B) of alpha-linolenic acid reference standard. Figure S9: MS data of (A) peak at Rt = 36.00 with a single charge state of MH-279.23 g/mol and (B) linoleic acid reference standard. Figure S10: Fragments (A) of 279.2 g/mol signal in fraction T-F5 and (B) of linoleic acid reference standard. Figure S11: MS data of (A) peak at Rt = 38.51 with a single charge state of MH-281.2 g/mol and (B) oleic acid reference standard. Figure S12: Fragments (A) of 281.2 g/mol signal in fraction T-F5 and (B) of oleic acid reference standard. Figure S13: IC_50_ values (µg/mL) of extracts (left of dashed line) and CPC fractions (right of dashed line) required to inhibit 0.1 mM DPPH. Table S1: Phytochemical composition in mg/g dry sample and relative COX-2 inhibition to control (%) of the Waltheria Indica leaf extracts.

## Sample Availability

Samples of *Waltheria indica* L. leaves are available from the authors.

## References

[1] Jones S.A. (2005). Directing Transition from Innate to Acquired Immunity: Defining a Role for IL-6. J. Immunol..

[2] Pasparakis M., Haase I., Nestle F.O. (2014). Mechanisms Regulating Skin Immunity and Inflammation. Nat. Rev. Immunol..

[3] Philpott M., Ferguson L.R. (2004). Immunonutrition and Cancer. Mutat. Res. Fundam. Mol. Mech. Mutagenesis.

[4] Greene E.R., Huang S., Serhan C.N., Panigrahy D. (2011). Regulation of Inflammation in Cancer by Eicosanoids. Prostaglandins Other Lipid Mediat..

[5] Mantovani A., Allavena P., Sica A., Balkwill F. (2008). Cancer-Related Inflammation. Nature.

[6] Leong J., Hughes-Fulford M., Rakhlin N., Habib A., Maclouf J., Goldyne M.E. (1996). Cyclooxygenases in Human and Mouse Skin and Cultured Human Keratinocytes: Association of COX-2 Expression with Human Keratinocyte Differentiation. Exp. Cell Res..

[7] Buckman S. (1998). COX-2 Expression Is Induced by UVB Exposure in Human Skin: Implications for the Development of Skin Cancer. Carcinogenesis.

[8] An K.P., Athar M., Tang X., Katiyar S.K., Russo J., Beech J., Aszterbaum M., Kopelovich L., Epstein E.H., Mukhtar H. (2007). Cyclooxygenase-2 Expression in Murine and Human Nonmelanoma Skin Cancers: Implications for Therapeutic Approaches. Photochem. Photobiol..

[9] Di Meglio P., Perera G.K., Nestle F.O. (2011). The Multitasking Organ: Recent Insights into Skin Immune Function. Immunity.

[10] Segal B.H., Leto T.L., Gallin J.I., Malech H.L., Holland S.M. (2000). Genetic, Biochemical, and Clinical Features of Chronic Granulomatous Disease. Medicine.

[11] Nathan C. (2002). Points of Control in Inflammation. Nature.

[12] Shishodia S., Potdar P., Gairola C.G., Aggarwal B.B. (2003). Curcumin (Diferuloylmethane) down-Regulates Cigarette Smoke-Induced NF-KappaB Activation through Inhibition of IkappaBalpha Kinase in Human Lung Epithelial Cells: Correlation with Suppression of COX-2, MMP-9 and Cyclin D1. Carcinogenesis.

[13] Han J.A., Kim J.-I., Ongusaha P.P., Hwang D.H., Ballou L.R., Mahale A., Aaronson S.A., Lee S.W. (2002). P53-Mediated Induction of Cox-2 Counteracts P53- or Genotoxic Stress-Induced Apoptosis. EMBO J..

[14] Shacter E., Weitzman S.A. (2002). Chronic Inflammation and Cancer. Oncology.

[15] Pockaj B.A., Basu G.D., Pathangey L.B., Gray R.J., Hernandez J.L., Gendler S.J., Mukherjee P. (2004). Reduced T-Cell and Dendritic Cell Function Is Related to Cyclooxygenase-2 Overexpression and Prostaglandin E2 Secretion in Patients With Breast Cancer. Ann. Surg. Oncol..

[16] Misra S., Hascall V.C., Markwald R.R., O’Brien P.E., Ghatak S., Turksen K. (2018). Inflammation and cancer. Wound Healing: Stem Cells Repair and Restorations, Basic and Clinical Aspects.

[17] Choy E., Rose-John S. (2017). Interleukin-6 as a Multifunctional Regulator: Inflammation, Immune Response, and Fibrosis. J. Scleroderma Relat. Disord..

[18] Zhuang Y., Lyga J. (2014). Inflammaging in Skin and Other Tissues—The Roles of Complement System and Macrophage. IADT.

[19] Desai S.J., Prickril B., Rasooly A. (2018). Mechanisms of Phytonutrient Modulation of Cyclooxygenase-2 (COX-2) and Inflammation Related to Cancer. Nutr. Cancer.

[20] Nirmala C., Sridevi M. (2021). Ethnobotanical, Phytochemistry, and Pharmacological Property of *Waltheria indica* Linn. Future J. Pharm. Sci..

[21] Zongo F., Ribuot C., Boumendjel A., Guissou I. (2013). Botany, Traditional Uses, Phytochemistry and Pharmacology of *Waltheria indica* L. (Syn. Waltheria Americana): A Review. J. Ethnopharmacol..

[22] Flatie T., Gedif T., Asres K., Gebre-Mariam T. (2009). Ethnomedical Survey of Berta Ethnic Group Assosa Zone, Benishangul-Gumuz Regional State, Mid-West Ethiopia. J. Ethnobiol. Ethnomed..

[23] Adjanohoun E., Adjakidje V., Ahyi M.R.A., Akoegninou A., d’Almeida J., Apovo F., Boukef K., Chadare M., Gusset G., Dramane K. (1989). Contribution aux études Ethnobotaniques et Floristiques en République Populaire du Bénin.

[24] Ruffo C.K. (1991). A Survey of medicinal plants in Tabora region, Tanzania. Traditional Medicinal Plants.

[25] Zerbo P., Millogo-Rasolodimey J., Nacoulma-Ouerdraogo O., Van Damme P. (2008). Contribution à La Connaissance Des Plantes Médicinales Utilisées Dans Les Soins Infantiles En Pays San, Au Burkina Faso. Int. J. Biol. Chem. Sci..

[26] Borokini T.I., Omotayo F.O. (2012). Phytochemical and Ethnobotanical Study of Some Selected Medicinal Plants from Nigeria. JMPR.

[27] Cretton S., Bréant L., Pourrez L., Ambuehl C., Perozzo R., Marcourt L., Kaiser M., Cuendet M., Christen P. (2015). Chemical Constituents from *Waltheria indica* Exert in Vitro Activity against Trypanosoma Brucei and T. Cruzi. Fitoterapia.

[28] Vedavathy S., Rao K.N. (1995). Anti-inflammatory activity of some indigenous medicinal plants of Chittor district, Andhra Pradesh. Indian Drugs.

[29] Yougbare-Ziebrou M.N., Lompo M., Ouedraogo N., Yaro B., Guissoun I.P. (2016). Antioxidant, Analgesic and Anti-Inflammatory Activities of the Leafy Stems of *Waltheria indica* L. (Sterculiaceae). J. Appl. Pharm. Sci..

[30] Chandekar A., Vyas A., Upmanyu N., Tripathi A., Agrawal S. (2017). Preliminary Screening of *Waltheria indica* (L.) Plant for Its Anti-Inflammatory Activity. Int. J. Phytomed..

[31] Owemidu I., Olubori M., Faborode O., Oloyede O., Onasanwo S. (2018). Anti-Nociceptive and Anti- Inflammatory Activities of the Methanol Extract of Waltheria Americana Leaf in Experimental Animals. J. Complement. Med. Res..

[32] Rao Y.K., Fang S.-H., Tzeng Y.-M. (2005). Inhibitory Effects of the Flavonoids Isolated from *Waltheria indica* on the Production of NO, TNF-a and IL-12 in Activated Macrophages. Biol. Pharm. Bull..

[33] Laczko R., Chang A., Watanabe L., Petelo M., Kahaleua K., Bingham J.-P., Csiszar K. (2020). Anti-Inflammatory Activities of Waltheria Indica Extracts by Modulating Expression of IL-1B, TNF-α, TNFRII and NF-ΚB in Human Macrophages. Inflammopharmacology.

[34] Monteillier A., Cretton S., Ciclet O., Marcourt L., Ebrahimi S.N., Christen P., Cuendet M. (2017). Cancer Chemopreventive Activity of Compounds Isolated from *Waltheria indica*. J. Ethnopharmacol..

[35] Carlsen I., Frøkiær J., Nørregaard R. (2015). Quercetin Attenuates Cyclooxygenase-2 Expression in Response to Acute Ureteral Obstruction. Am. J. Physiol. Ren. Physiol..

[36] Grochowski D.M., Locatelli M., Granica S., Cacciagrano F., Tomczyk M. (2018). A Review on the Dietary Flavonoid Tiliroside. Compr. Rev. Food Sci. Food Saf..

[37] De Paula Vasconcelos P.C., Seito L.N., Di Stasi L.C., Akiko Hiruma-Lima C., Pellizzon C.H. (2012). Epicatechin Used in the Treatment of Intestinal Inflammatory Disease: An Analysis by Experimental Models. Evid.-Based Complement. Altern. Med..

[38] Mutoh M., Takahashi M., Fukuda K., Komatsu H., Enya T., Matsushima-Hibiya Y., Mutoh H., Sugimura T., Wakabayashi K. (2000). Suppression by Flavonoids of Cyclooxygenase-2 Promoter-Dependent Transcriptional Activity in Colon Cancer Cells: Structure-Activity Relationship. Jpn. J. Cancer Res..

[39] Attiq A., Jalil J., Husain K., Ahmad W. (2018). Raging the War Against Inflammation With Natural Products. Front. Pharmacol..

[40] Termer M., Carola C., Salazar A., Keck C.M., Hemberger J., von Hagen J. (2021). Identification of Plant Metabolite Classes from *Waltheria indica* L. Extracts Regulating Inflammatory Immune Responses via COX-2 Inhibition. J. Ethnopharmacol..

[41] Lu Y., Luthria D. (2014). Influence of Postharvest Storage, Processing, and Extraction Methods on the Analysis of Phenolic Phytochemicals. Instrumental Methods for the Analysis and Identification of Bioactive Molecules.

[42] Ringbom T., Huss U., Stenholm Å., Flock S., Skattebøl L., Perera P., Bohlin L. (2001). COX-2 Inhibitory Effects of Naturally Occurring and Modified Fatty Acids. J. Nat. Prod..

[43] Sato I., Kofujita H., Tsuda S. (2007). Identification of COX Inhibitors in the Hexane Extract of Japanese Horse Chestnut (*Aesculus Turbinata*) Seeds. J. Vet. Med. Sci..

[44] Laneuville O., Breuer D.K., Xu N., Huang Z.H., Gage D.A., Watson J.T., Lagarde M., DeWitt D.L., Smith W.L. (1995). Fatty Acid Substrate Specificities of Human Prostaglandin-Endoperoxide H Synthase-1 and -2. J. Biol. Chem..

[45] Rieke C.J., Mulichak A.M., Garavito R.M., Smith W.L. (1999). The Role of Arginine 120 of Human Prostaglandin Endoperoxide H Synthase-2 in the Interaction with Fatty Acid Substrates and Inhibitors. J. Biol. Chem..

[46] Smith W.L., Malkowski M.G. (2019). Interactions of Fatty Acids, Nonsteroidal Anti-Inflammatory Drugs, and Coxibs with the Catalytic and Allosteric Subunits of Cyclooxygenases-1 and -2. J. Biol. Chem..

[47] Singleton V.L., Orthofer R., Lamuela-Raventós R.M. (1999). [14] Analysis of total phenols and other oxidation substrates and antioxidants by means of folin-ciocalteu reagent. Methods in Enzymology.

[48] Oludemi T., Barros L., Prieto M.A., Heleno S.A., Barreiro M.F., Ferreira I. (2018). Extraction of Triterpenoids and Phenolic Compounds from Ganoderma Lucidum: Optimization Study Using the Response Surface Methodology. Food Funct..

[49] Baccou J.C., Lambert F., Sauvaire Y. (1977). Spectrophotometric Method for the Determination of Total Steroidal Sapogenin. Analyst.

[50] Ncube B., Ngunge V.N., Finnie J.F., Van Staden J. (2011). A Comparative Study of the Antimicrobial and Phytochemical Properties between Outdoor Grown and Micropropagated Tulbaghia Violacea Harv. Plants. J. Ethnopharmacol..

[51] Wang Y., McAllister T.A. (2010). A Modified Spectrophotometric Assay to Estimate Deglycosylation of Steroidal Saponin to Sapogenin by Mixed Ruminal Microbes. J. Sci. Food Agric..

