# Supplementary material for: Activity-Guided Characterization of COX-2 Inhibitory Compounds in Waltheria indica L. Extracts
Source: Molecules. 2021 Nov 29;26(23):7240. doi: 10.3390/molecules26237240 (PMC8658768; doi:10.3390/molecules26237240)
Supplement: Supplementary file 1 [file molecules-26-07240-s001.zip › molecules-1474137 supplementary.pdf]

SUPPLEMENTARY MATERIAL

Activity-Guided Characterization of COX-2 Inhibitory Compounds in *Waltheria Indica L.* Extracts

Michael Termer [1,*], Christophe Carola [2], Andrew Salazar [2], Cornelia M. Keck [1], Juergen Hemberger [3] and Joerg von Hagen [2,3]

1 Department of Pharmaceutics and Biopharmaceutics, Philipps-University of Marburg, Robert-Koch-Str. 4, 35032 Marburg, Germany; cornelia.keck@pharmazie.uni-marburg.de
2 Merck KGaA, Frankfurterstr. 250, 64293 Darmstadt, Germany; christophe.carola@merckgroup.com (C.C.); andrew.salazar@merckgroup.com (A.S.); joerg.von.hagen@merckgroup.com (J.v.H.)
3 Department of Life Science Engineering, Institute for Biochemical Engineering & Analytics, University of Applied Sciences, Wiesenstr. 14, 35390 Giessen, Germany; juergen.hemberger@lse.thm.de
* Correspondence: michael.termer@pharmazie.uni-marburg.de

2.1 Centrifugal Partition Chromatography Fractionation

The chromatograms of the individual CPC fractions are summarized in Figure S1-5. The CPC fraction L–F2 primarily combines the substances up to Rt = 8 min (Figure S1).

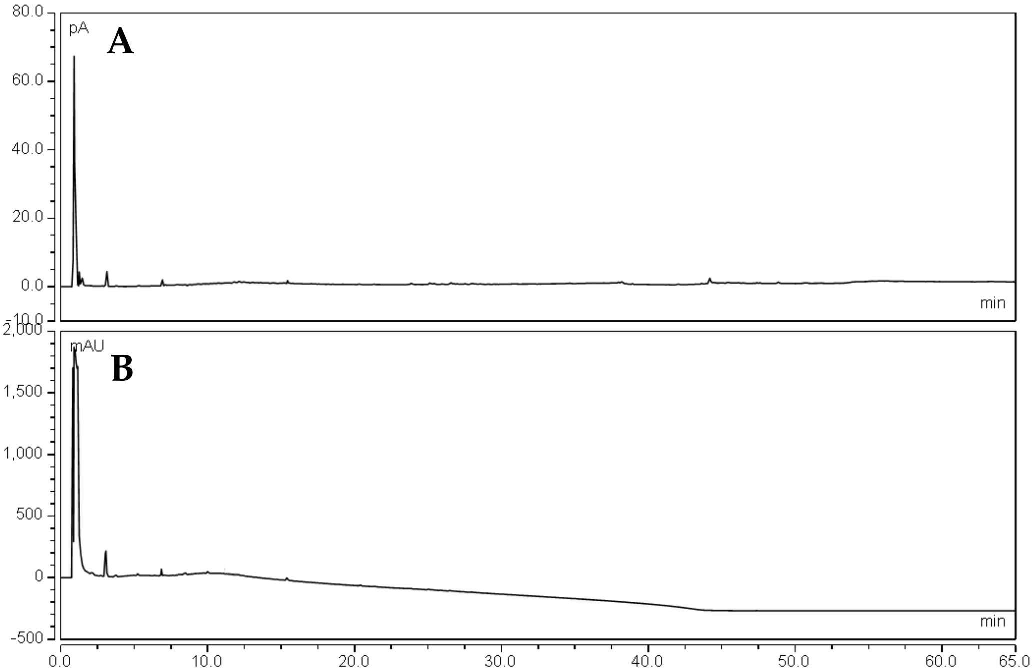

Figure S1: UHPLC chromatogram of CPC fraction L-F2 with (**A**) CAD and (**B**) DAD at 200 nm.

The fraction L-F3 contained less polar compounds up to Rt = 19 min including tiliroside, which was not present in L-F2 (Figure S2).

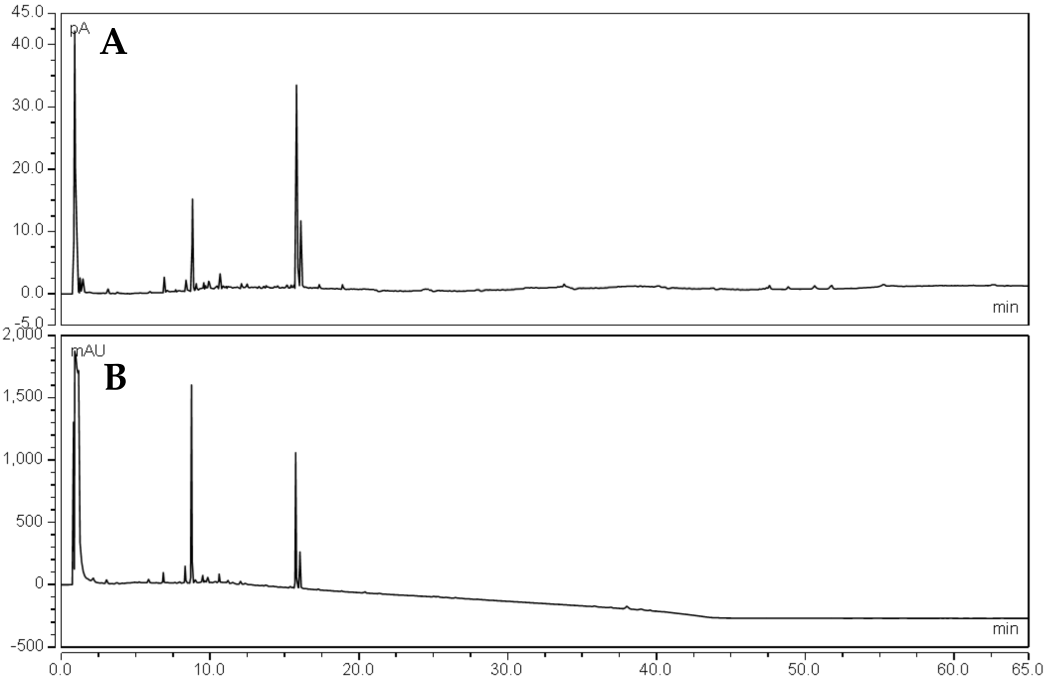

Figure S2: UHPLC chromatogram of CPC fraction L-F3 with **(A**) CAD and (**B**) DAD at 200 nm.

The fraction T-F2 contained substances between Rt = 0 and Rt = 19 min (Figure S3).

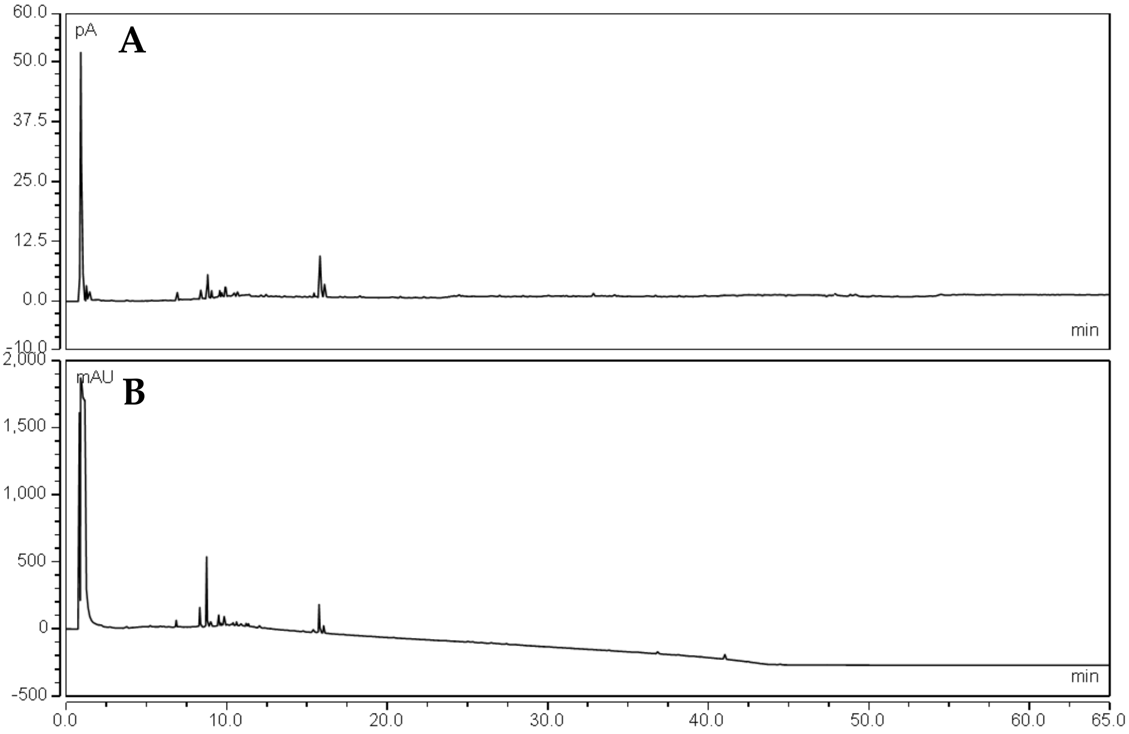

Figure S3: UHPLC chromatogram of CPC fraction T-F2 with **(A**) CAD and (**B**) DAD at 200 nm.

The fraction T-F5 collect molecules after Rt = 25 min up to Rt = 47 min (Figure S4).

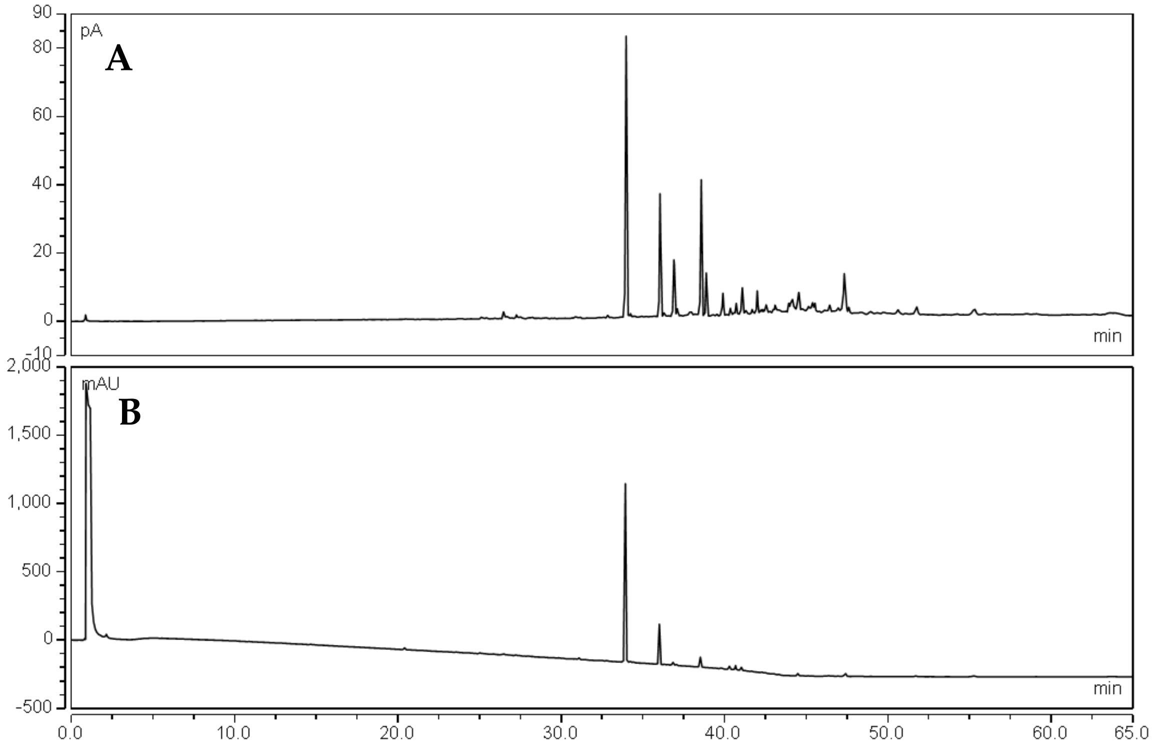

Figure S4: UHPLC chromatogram of CPC fraction T-F5 with **(A**) CAD and (**B**) DAD at 200 nm.

The CPC fraction T-F6 collect molecules from Rt = 33.5 min (Figure S5).

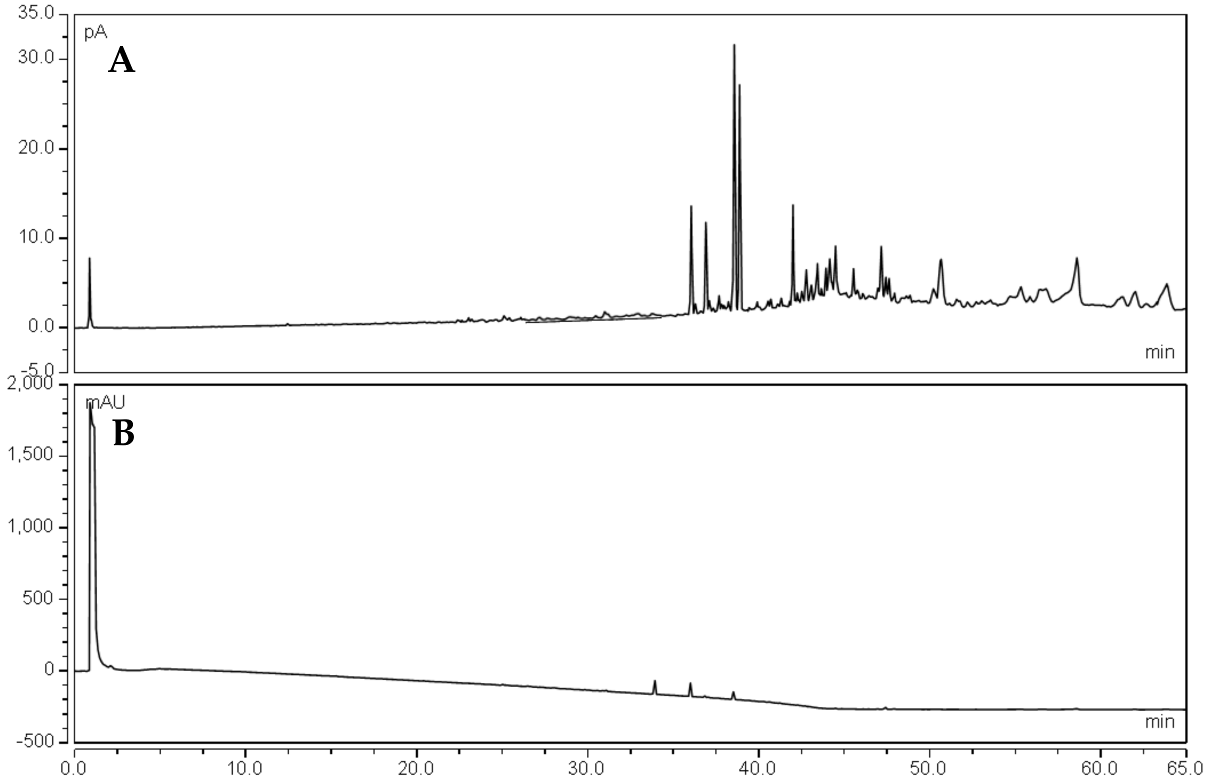

Figure S5: UHPLC chromatogram of CPC fraction T-F6 with **(A**) CAD and (**B**) DAD at 200 nm.

2.3 Identification of Fatty Acids in CPC Fraction T-F5

The identification of the fatty acids alpha-linolenic acid, linoleic acid and oleic acid in the CPC fraction T–F5 was carried out with Vanquish HPLC system coupled with high resolution mass spectrometer (Q Exactive™ Hybrid Quadrupol-Orbitrap™, Thermo Scientific™, USA) with H-ESI interface and negative ionization at 30 °C column temperature, 0.4 ml/min flow speed and 1 µL injection volume.

The separation was accomplished using C-18 column (ThermoFisher™ Hypersil Gold™ aQ, 150 × 2.1 mm, 1.9 µm) and gradient elution with solvent A: water/formic acid 99.9/0.1 (*v*/*v*) and B: acetonitrile/formic acid 99.9/0.1 (*v*/*v*). Gradient started at 5% (*v*/*v*) B holding for 1 min, ramping up to 50 % over 20 min, ramping up to 98 % over 20 min and holding for 25 min.

UV-HPLC chromatogram at 200 nm of the fraction T–F5 showed three intense UV peaks at Rt = 33.91 min, R_t = 36.00 min and R_t = 38.51 min (Figure S6, A), which corresponds to reference standards of alpha-linolenic acid (Figure S6, B), linoleic acid (Figure S6, C) and oleic acid (Figure S6, D).

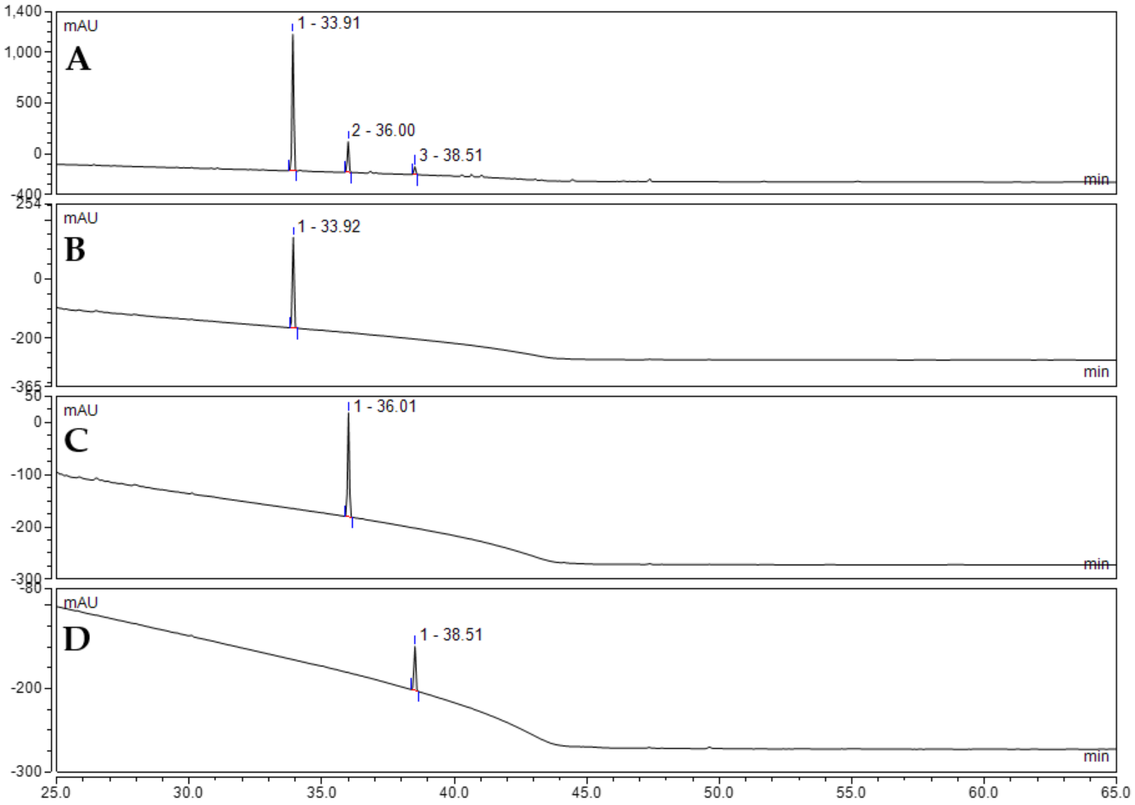

Figure S6: UHPLC-UV chromatogram at 200 nm of **(A)** CPC fraction T-F5 (2 mg/mL) and reference standards **(B)** alpha-linolenic acid (0.05 mg/mL), **(C)** linoleic acid (0.05 mg/mL), **(D)** oleic acid (0.05 mg/mL).

MS data show peak at R_t = 33.91 min with a single charge state of MH-277.2 g/mol (Figure S7, A), which corresponds to that of alpha-linolenic acid reference standard (Figure S7, B)

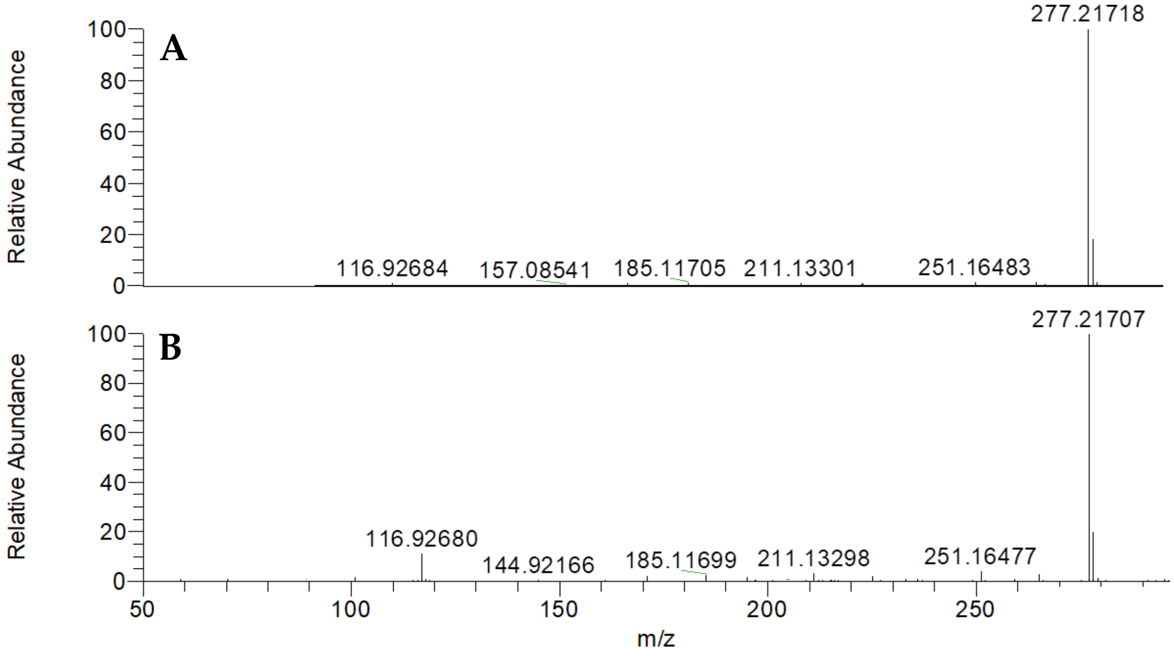

Figure S7: MS data of **(A)** peak at R_t = 33.91 with a single charge state of MH-277.2 g/mol and **(B)** alpha-linolenic acid reference standard.

Fragments of the 277.2 g/mol signal in fraction T-F5 (Figure S8, A) correspond to alpha-linolenic acid reference standard fragments (Figure S8, B).

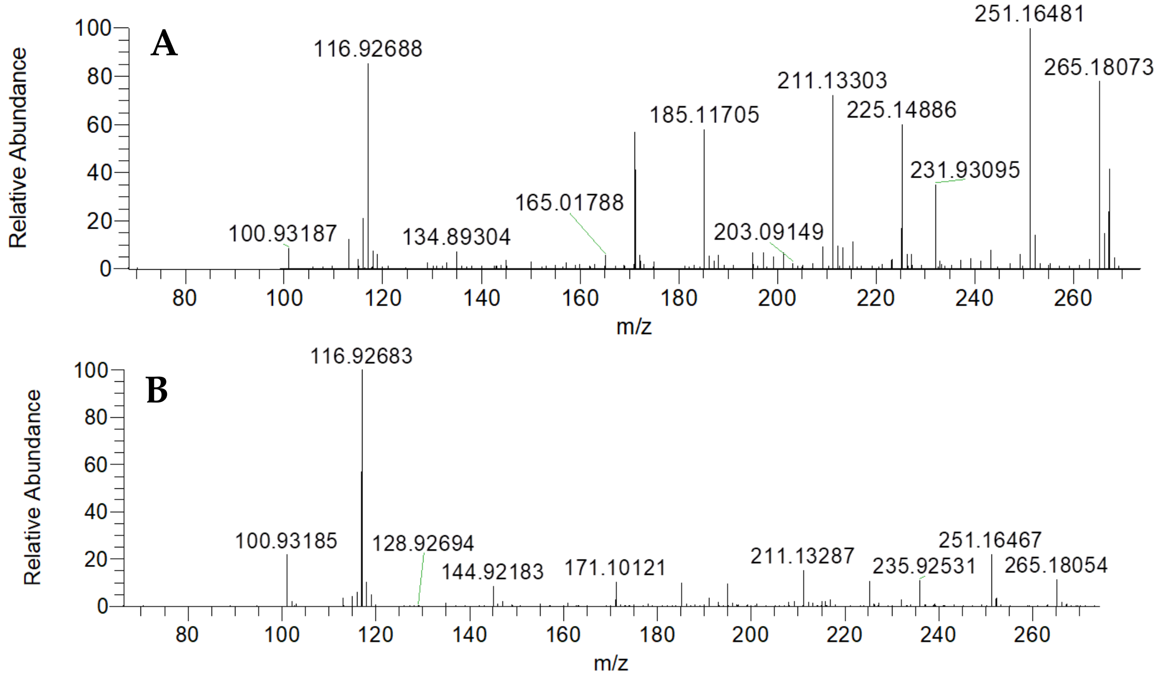

Figure S8: Fragments **(A)** of 277.2 g/mol signal in fraction T-F5 and **(B)** of alpha-linolenic acid reference standard.

MS data for the peak at R_t = 36.00 min with a single charge state of MH-279.2 g/mol (Figure S9, A), which corresponds to that of linoleic acid reference standard (Figure S9, B).

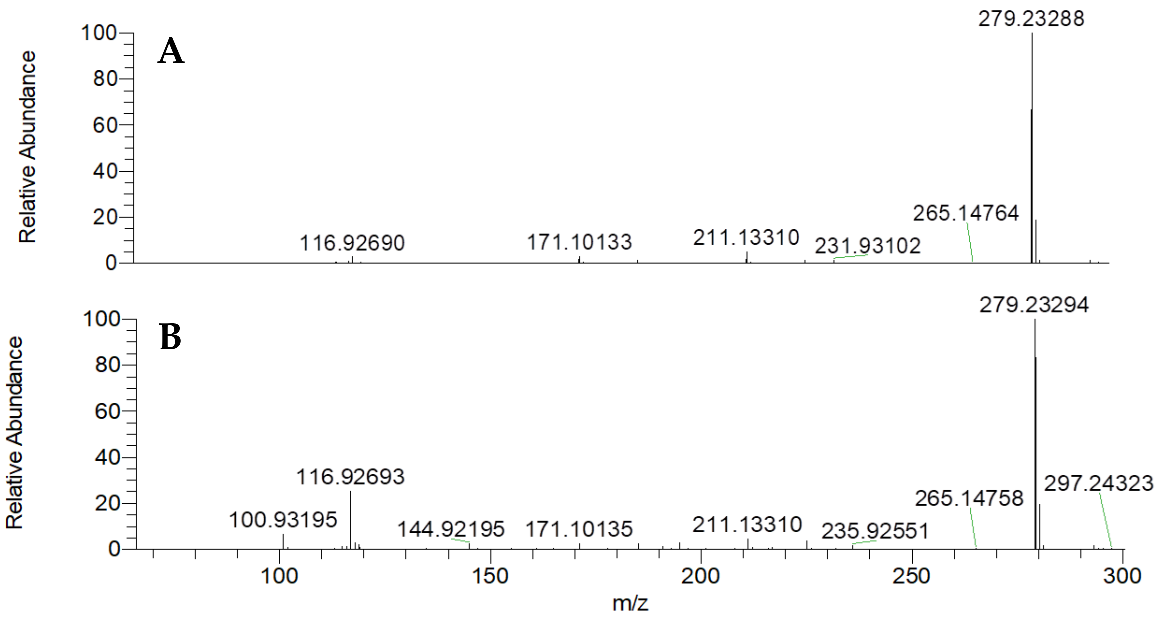

Figure S9: MS data of **(A)** peak at R_t = 36.00 with a single charge state of MH-279.23 g/mol and **(B)** linoleic acid reference standard.

Fragments of the 279.2 g/mol signal in fraction T-F5 (Figure S10, A) correspond to linoleic acid reference standard fragments (Figure S10, B).

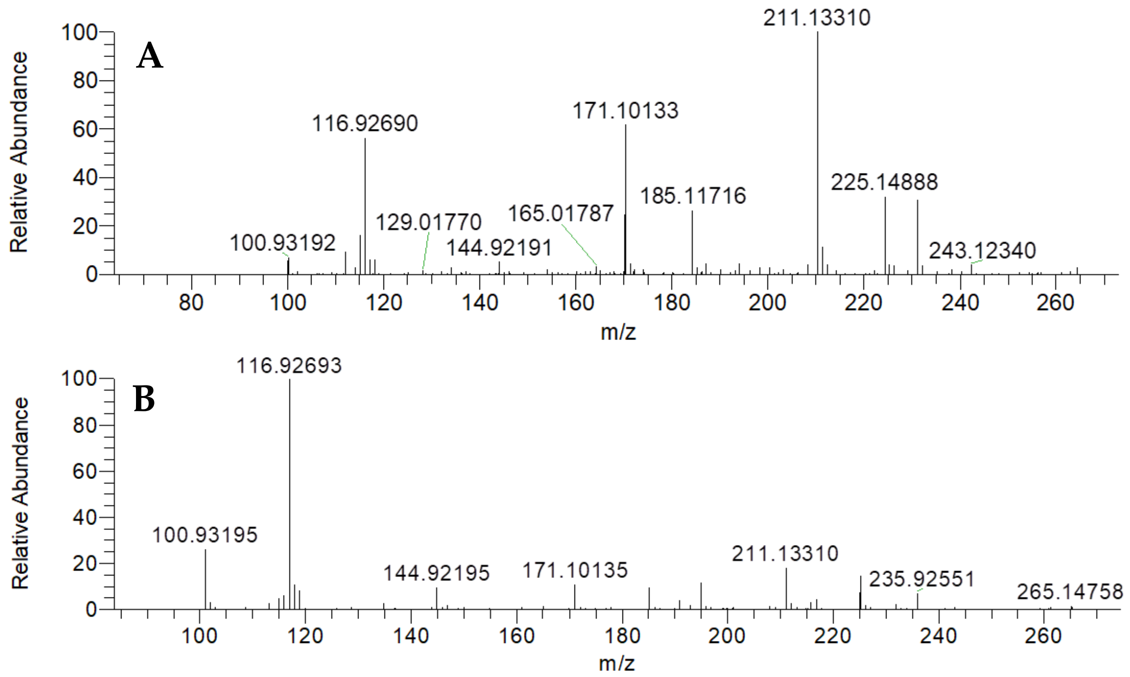

Figure S10: Fragments **(A)** of 279.2 g/mol signal in fraction T-F5 and **(B)** of linoleic acid reference standard.

MS data for the peak at R_t = 38.51 min with a single charge state of MH-281.2 g/mol (Figure S11, A), which corresponds to that of oleic acid reference standard (Figure S11, B)

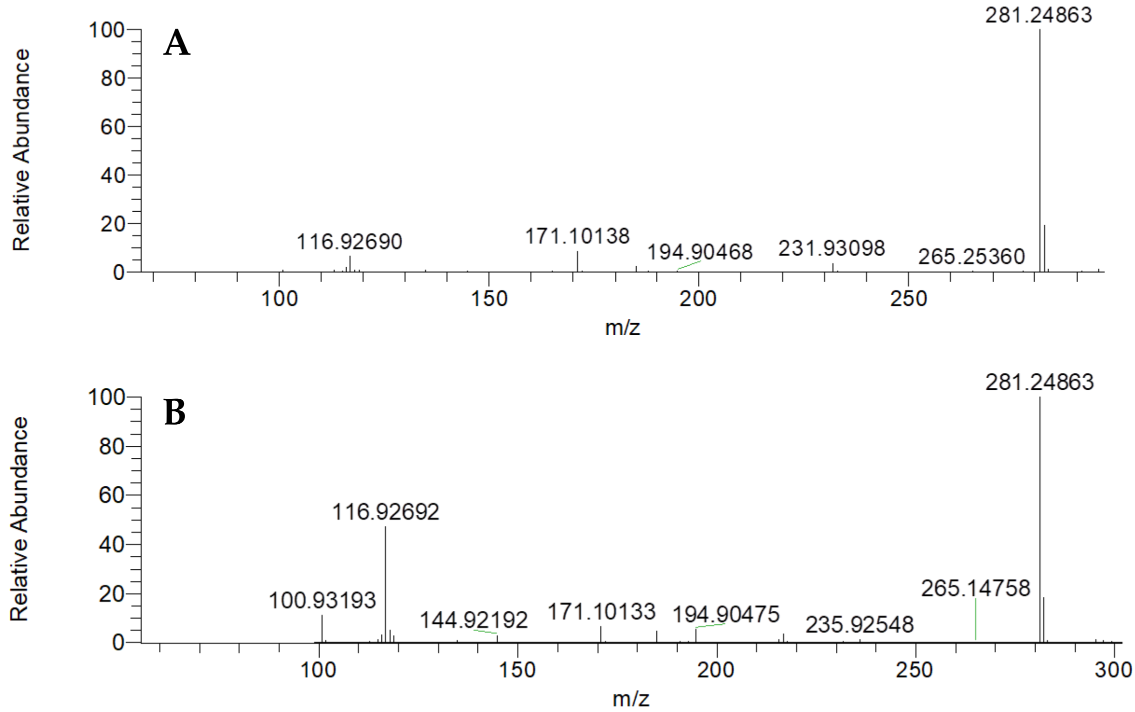

Figure S11: MS data of **(A)** peak at R_t = 38.51 with a single charge state of MH-281.2 g/mol and **(B)** oleic acid reference standard.

Fragments of the 281.2 g/mol signal in fraction T-F5 (Figure S12, A) correspond to oleic acid reference standard fragments (Figure S12, B).

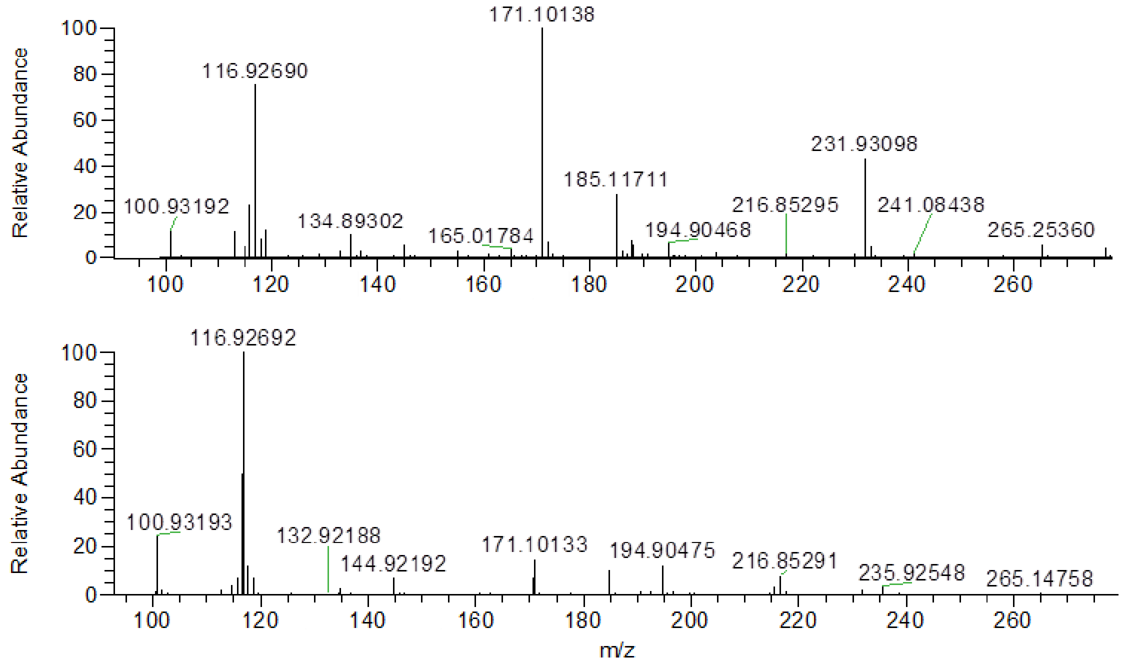

Figure S12: Fragments **(A)** of 281.2 g/mol signal in fraction T-F5 and **(B)** of oleic acid reference standard.

2.6. Antioxidative Potential of Extracts and CPC Fractions

The antioxidant potential of the extracts and CPC fractions was assessed based on their concentration required to inhibit 50 % (IC_{50}) of 0.1 mM 1,1-diphenyl-2-picrylhydrazyl radicals (DPPH). The IC_{50} values were calculated using a calibration curve with five data points with the respective sample (0.050–0.50 mg/mL) after determining the inhibition of the 0.1 mM DPPH solution for the respective concentration. Sample solution (1.0 mL) for the respective concentration was mixed with DPPH solution (9 mL; 0.112 mM dissolved in ethanol) resulting in sample (S) reacting with 0.10 mM DPPH. After 60 min reaction time the reduction of DPPH radicals was measured by the decrease in absorption (A) at 515 nm using a UV–VIS spectrophotometer (Varian Cary 60, Agilent). Ethanolic 0.1 mM DPPH solution was used as control sample (CS). The inhibition of 0.1 mM DPPH for the respective sample was calculated following equation S1.

$$\% \text{ Inhibition of 0.1 mM DPPH} = (A(Cs)-A(s)) / A(Cs) \times 100 \qquad (S1)$$

After determining the DPPH inhibition for each sample following equation 1, the IC_{50} values were calculated based on the calibration curves generated from these data and summarized in Figure S13. All assays were performed in triplicate and results expressed as IC_{50} in mg/mL.

The obtained results demonstrated that among the extracts, EA90 had lowest antioxidant potential with IC_{50} = 108 µg/mL and E70 had the highest with IC_{50} = 37 µg/mL. Extracts E30, E90, E150, M90, and W90 exhibited significantly higher antioxidant potential relative to EA90 with IC_{50} values of 48, 41, 45, 43, and 44 µg/mL, respectively. Among the CPC fractions, the polar fractions L-F2, L-F3, and T-F2 with IC_{50} values of 24.5, 31.5, and 25.4 µg/mL, respectively, showed significantly stronger antioxidant potential than the nonpolar fractions T-F5 and T-6 with IC_{50} values of 617 and 362 µg/mL, respectively.

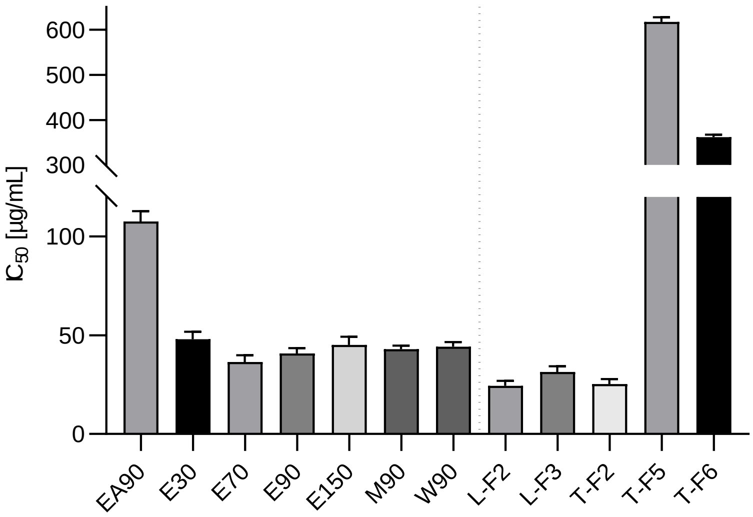

Figure S13: IC_{50} values (µg/mL) of extracts (left of dashed line) and CPC fractions (right of dashed line) required to inhibit 0.1 mM DPPH. Data points represent the mean value ± standard deviations of three samples (*n* = 3).

Given that samples with the highest COX-2 inhibition activity (EA90 for extracts and T-F5, T-F6 for CPC fractions) possess the weakest antioxidant capacity, it became evident that the observed COX-2 inhibition was not driven by antioxidants.

2.8 COX-2 Inhibition Prediction Model

To predict the COX-2 inhibitory properties of different *Waltheria indica* extracts, two mathematical models (model A and B) were generated, considering Steroidal- and Triterpenoid saponins as well as ALA and LA as positive contributors to the COX-2 inhibition. The phytochemical composition of the investigated extracts and their COX-2 inhibition activity served as the data basis (Table S1)

Table S1: Phytochemical composition in mg/g dry sample and relative COX-2 inhibition to control (%) of the Waltheria Indica leaf extracts. GAE = Gallic acid equivalent; OAE = Oleanolic acid equivalent; DE = Diosgenin equivalent. EA90, 90 °C ethyl acetate; E30-E70-E90-E150, 30-70-90-150 °C ethanol; M90, 90 °C methanol; W90, 90 °C water; n.d., not detectable. Values are means ± standard deviations of triplicate measurements.

Sample	Triterpenoid-saponins [mg OAE/g]	Steroidal-saponins [mg DE/g]	Alpha-Linolenic acid [mg/g]	Linoleic acid [mg/g]	COX-2 inhibition [%]
EA90	195.0 ± 3.6	113.6 ± 2.7	40.1 ± 1.5	26.7 ± 1.1	75.3 ± 4.0
E30	214.8 ± 2.3	85.1 ± 1.3	27.1 ± 1.5	17.6 ± 0.9	68.0 ± 2.7
E70	182.8 ± 5.3	62.3 ± 3.5	17.0 ± 1.6	10.7 ± 0.7	57.3 ± 4.3
E90	143.6 ± 2.5	57.7 ± 1.5	13.1 ± 1.4	8.0 ± 0.4	56.0 ± 2.6
E150	120.1 ± 3.6	59.2 ± 1.4	10.0 ± 0.4	5.9 ± 0.3	52.3 ± 5.9
M90	106.0 ± 3.9	53.0 ± 0.8	7.3 ± 0.4	4.3 ± 0.2	45.7 ± 3.1
W90	47.3 ± 2.1	7.4 ± 0.7	0	0	23.7 ± 0.7

Identification of Tiliroside in Waltheria Indica Extract

Identification of tiliroside in *Waltheria Indica* extracts were carried out on Vanquish HPLC system coupled with Thermo Fusion™ Orbitrap Tribrid™ mass spectrometer (Thermo Scientific™) with H-ESI interface and positive ionization. Separation was accomplished using C-18 column (Thermo Acclaim™ 120, 100 × 4.6 mm, 5 µm) and gradient elution with solvent A: water/acetonitrile/trifluoroacetic acid 95/5/0.125 (*v*/*v*/*v*) and B: water/acetonitrile/trifluoroacetic acid 5/95/0.125 (*v*/*v*/*v*). Gradient started at 7% (*v*/*v*) B, ramping up to 28 % over 15 min, ramping up to 56 % over 5 min, further to 95 % over 5 min, held for 3 min and ramping down to 7 % over 8 min. UV detection was performed at 315 nm, 25 °C column temperature, 1.5 ml/min flow speed and 10 µL injection volume. UV-HPLC profile of *Waltheria Indica* extract obtained with ethanol at 90 °C show an intense UV peak at R_t = 14.47 min (**Figure S14**), which corresponds to tiliroside standard (data not shown).

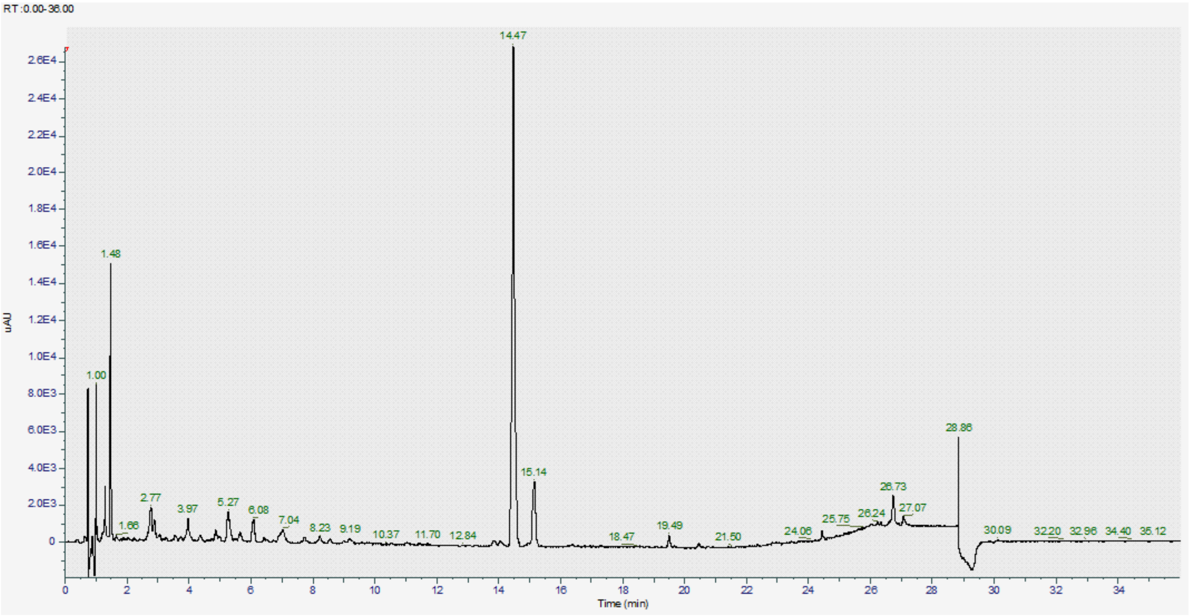

Figure S14: UV-HPLC profile at 315 nm of *Waltheria Indica* extract obtained with ethanol at 90 °C.

MS data in **Figure S15** of the peak at R_t = 14.47 show a single charge state MH+ 595.1432 g/mol and MNa+617.1249 g/mol, which corresponds to that of tiliroside.

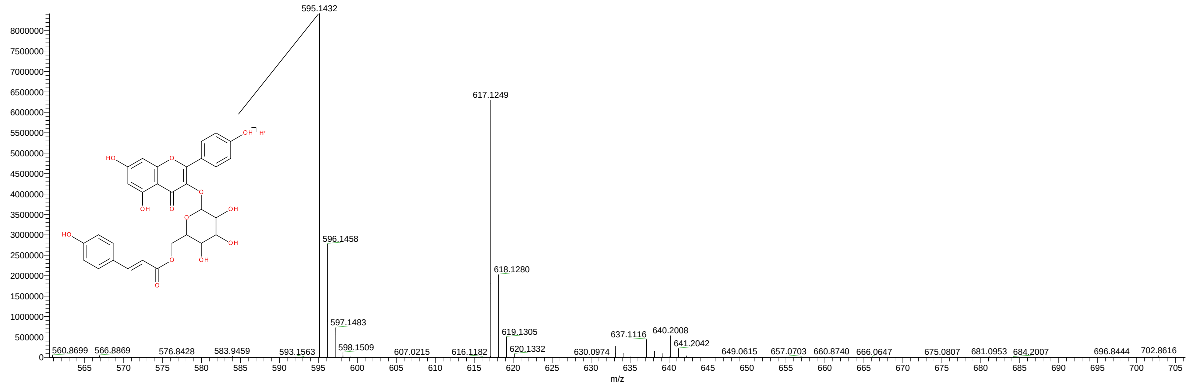

Figure S15: MS data of the peak at R_t = 14.47.

Fragments of 594.1432 g/mol signal correspond to tiliroside fragments (**Figure S16**).

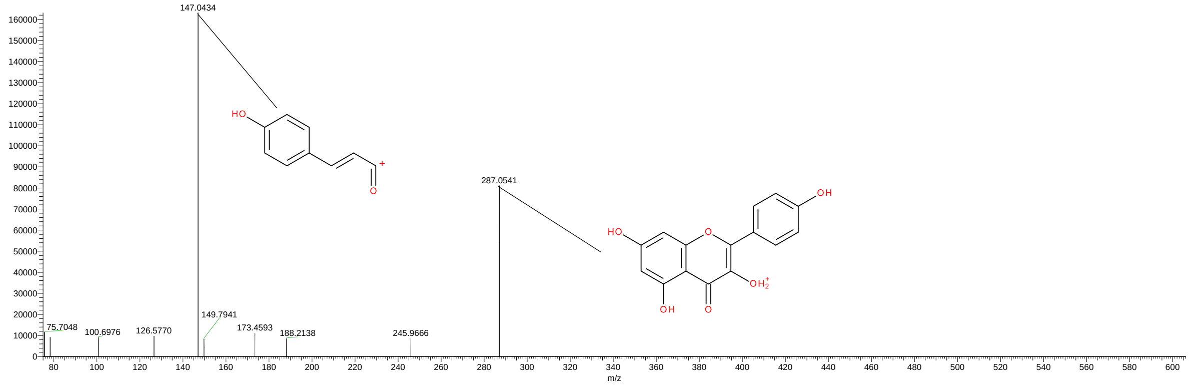

Figure 16: Fragments of 595.1432 g/mol signal with their corresponding structures.
